# Conceptualizing and Measuring Support for Collective Violence

**DOI:** 10.1002/ab.70063

**Published:** 2026-02-22

**Authors:** Ramzi Abou‐Ismail, Joseph B. Phillips, Aleksandra Cichocka, Hakan Çakmak, Sami Çoksan, Nikhil K. Sengupta

**Affiliations:** ^1^ School of Arts and Sciences ‐ Department of Political and International Studies Lebanese American University Beirut Lebanon; ^2^ School of Law & Politics Cardiff University Cardiff Wales; ^3^ School of Psychology University of Kent Canterbury England; ^4^ Department of Social Psychology University of Groningen Groningen Netherlands; ^5^ Department of Psychology Western University London Canada

**Keywords:** collective violence, intergroup conflict, measurement, scale development, social identity

## Abstract

Although collective violence remains a pervasive issue affecting many societies today, the specific psychological mechanisms underlying individual differences in support for collective violence are relatively understudied. In four studies, using five samples from Lebanon, Syria, and Turkey (total *N* = 3758), we conceptualize and develop a new multidimensional scale for measuring individual differences in collective violence beliefs. Contrary to some prior theorizing and extant research on interpersonal violence, we found that people's justifications for collective violence are structured based on the target of the act rather than the intensity of the violent act. Consequently, we developed and validated a Two‐Dimensional Collective Violence Beliefs Scale (CVBS: 2D) that distinguishes between violence targeted at outgroup members, referred to as diffuse collective violence, and violence targeted at outgroup leaders, which we term upward collective violence. We validate this novel scale in multiple contexts and discuss the implications of the two dimensions of collective violence revealed in this study.

## 1 Introduction

Collective violence, an ancient phenomenon, and a contemporary reality, continues to afflict many societies (Allen and Anderson 2017; Anderson and Bushman 2002; Gómez et al. 2016). While social science research on collective violence has primarily focused on structural causes (see Dixon 2009), particularly in political science (Balcells and Stanton 2021; Fearon and Laitin 2003) and economics (Collier 2004), studies exploring the proximal, psychological factors that produce individual differences in support for collective violence (e.g., beliefs and dispositions) are limited (Bartusevičius et al. 2020; Kalmoe and Mason 2022). Most of this research treats collective violence as a unidimensional concept (e.g., Setiawan et al. 2020; Spanovic et al. 2010; Tausch et al. 2011), and a standard approach to conceptualizing and measuring individuals' support for collective violence within intergroup relations is lacking, despite recent attempts (Kalmoe and Mason 2022; Westwood et al. 2022; Winiewski and Bulska 2020).

There is also an extensive body of research in conflict resolution studies, which investigates attitudes toward collective violence through data collected in conflict zones. Studies such as those by Dyrstad and Hillesund (2020) and Koos (2018) have significantly contributed to our understanding of how various grievances—economic, political, collective, and individual—drive support for violence. These studies highlight the importance of considering the specific contexts and multifaceted motivations behind support for violence, which aligns with our approach of distinguishing between different dimensions of collective violence.

In this article, we review recent developments in the conceptualization and measurement of collective violence beliefs within intergroup contexts and draw upon the more comprehensive literature on interpersonal violence (Parrott and Giancola 2007) to develop and test a new multidimensional scale for collective violence beliefs. In our examination, this scale's relationship to other theoretically pertinent constructs and its functionality under varying levels of perceived threat are explored. Recognizing the complexities of threat perception, we acknowledge that while direct research is limited, psychological, sociopolitical, and socioeconomic theories often imply threat as a factor in collective violence, albeit not universally required (see Anderson and Bushman 2002; Esteban et al. 2012; Kalmoe and Mason 2022). The scale's validation in diverse contexts, such as Lebanese sectarianism, Syrian pro‐vs‐anti‐Assad, and Turkish‐Kurdish politics, provides critical insights into the multifaceted nature of collective violence beliefs.

### 1.1 Collective Violence

Buss (1961) provided a seminal and widely cited definition of aggression as “a response that delivers noxious stimuli to another organism.” Subsequent definitions (Anderson and Bushman 2002; Berkowitz 1993; Bushman and Anderson 2001; Meloy 2006) have retained the core theme while addressing the long‐term goal and intentionality of the act. Literature often differentiates between violence and aggression, with the latter including less extreme forms of violence (Anderson and Bushman 2002). We propose that aggression and violence differ in degree rather than kind, as both involve intentional harm. However, it remains an empirical question whether less severe acts differ qualitatively from more severe ones, which we will investigate in the current studies.

Collective violence, a specific instance of violence, occurs within intergroup contexts. Some scholars define collective violence as a violent act committed on behalf of a group and sanctioned by the group (Littman and Paluck 2015), while others argue it requires involvement and coordination of two or more individuals (Tilly 2003; Winiewski and Bulska 2020). However, both definitions exclude certain common occurrences. Some people may commit a violent act on behalf of a group identity even if group leaders do not sanction it. Some people commit these acts alone, yet explicitly tie their act to defending the wellbeing and rights of a specific group.

To accommodate each of these events, we adopt a definition of collective violence as any violent act conducted by one or more group‐identifying members acting on behalf of the group, intending to cause harm to an outgroup, with or without the ingroup's consent, approval, or knowledge. This aligns with intergroup relations literature, which views any interaction between individuals as an intergroup dynamic if they represent their respective groups (Tajfel et al. 1979). With this definition of collective violence in mind, we now explore conceptualizing and measuring beliefs about (and/or attitudes towards) collective violence at the individual level, taking into account their role in personal and intergroup beliefs as well as intergroup relations.

### 1.2 The Nature and Measurement of Collective Violence Beliefs

Social psychological research on collective violence is still emerging, and a major issue constraining this research is practicality. While studies on interpersonal violence often rely on incarcerated populations known to have varying latent propensities for interpersonal violence, capturing variation in collective violence may necessitate samples from contexts with a higher probability of violent intergroup conflict. This can be dangerous to obtain and ethically challenging (Winiewski and Bulska 2020).

To address practical concerns, we can identify and assess individuals who are more likely to support collective violence in settings that may not currently be experiencing armed conflict, but still have high levels of intergroup tensions, various degrees of intergroup conflict, or a history of endorsing aggressive behavior. Studies such as those by Uexkull et al. (2020) and Linke et al. (2015) underscore the importance of considering environmental stressors and public attitudes towards violence in conflict‐affected regions. Therefore, focusing on non‐Western Educated Industrial Rich, and Democratic (WEIRD), understudied contexts, which are often conflict‐affected regions and feature intergroup tensions, enables a deeper understanding of the human condition and the complexities of collective violence across diverse populations (see Newson et al. 2019). By examining these individuals in diverse settings, we could better understand the factors that contribute to collective violence beliefs.

In addressing the construct of collective violence, it is essential to consider potential issues such as the reluctance to admit illegal acts, which may hinder honest responses when asking about behaviors or intentions. One alternative approach is to focus on measuring attitudinal associations with collective violence (Kalmoe and Mason 2022), which may provide insights into individuals' predispositions without explicitly inquiring about their intentions or willingness to engage in acts themselves. It is important to note that attitudinal measures are not intended to be in opposition to existing measures of behavioral intentions. Rather, we view these attitudinal measures as complementary to the literature. By assessing both attitudes and behavioral intentions, we can gain a more comprehensive understanding of the factors that contribute to collective violence beliefs. Our aim is to provide additional insights that can be used alongside existing measures of intentions to further advance the field and develop a more holistic understanding of collective violence.

Existing research on collective violence in social psychology primarily assesses its attitudinal dimension (Setiawan et al. 2020; Spanovic et al. 2010; Subagya 2015), often treating collective violence as unidimensional with some exceptions (e.g., Winiewski and Bulska 2020). However, understanding the dimensionality of collective violence is crucial because different dimensions are motivated by different constructs, as demonstrated by Winiewski and Bulska (2020). Their research found that distinct types of collective violence are influenced by varying predictors, highlighting the necessity to consider multiple dimensions. Similarly, in the context of interpersonal violence, different types of violence are driven by different predictors (see Parrott and Giancola 2007 for a full review). This multidimensional perspective allows for a more nuanced understanding of the underlying factors and motivations.

In our current research, we assess the dimensionality of collective violence and develop a scale that adequately captures its multifaceted nature. We focus on the target of the act, distinguishing between violence targeted at outgroup members (diffuse collective violence) and violence targeted at outgroup leaders (upward collective violence). The justification for the upward collective violence dimension is supported by extensive research on violence towards the state and its representatives. Studies such as those by Dyrstad and Hillesund (2020), Koos (2018), and Jaeger et al. (2015) highlight how political grievances and dissatisfaction with governance can drive support for violence against state leaders, reflecting broader patterns of upward collective violence. Conversely, the diffuse collective violence dimension aligns with the standard approach of examining violence or prejudice against outgroup members, commonly found in studies of intergroup relations and prejudice (e.g., Setiawan et al. 2020; Spanovic et al. 2010). By incorporating this broader range of dimensions, we aim to develop a more comprehensive understanding of the factors that contribute to individual differences in support for collective violence.

Moreover, in designing scales to measure collective violence beliefs, we strive to make them more generalizable by avoiding specific stimuli tied to a particular context. By doing so, we can ensure that our measurements are applicable across different populations and intergroup dynamics (Hewstone et al. 2002). This would enable a more robust examination of the underlying psychological processes that drive collective violence beliefs and facilitate comparisons between different contexts and populations.

### 1.3 A Refined Approach to Measuring Collective Violence Beliefs

While Winiewski and Bulska (2020) conceptualize collective violence as primarily a response to salient intergroup threats, our findings suggest a more nuanced perspective. We argue that individuals' perceptions of violence as a legitimate tool for achieving ingroup political objectives can vary independently of their current sense of threat. This viewpoint aligns with some theoretical frameworks in psychology and socio‐politics, recognizing that the propensity for violence is not solely reactionary but may be deeply embedded in ideological beliefs and group dynamics (see Anderson and Bushman 2002; Meloy 2006; Tilly 2003). This understanding underscores the importance of considering both the immediate context and the underlying belief systems when analyzing support for collective violence. Our approach allows us to assess these beliefs in the absence of a salient threat. Our perspective aligns with other intergroup belief systems, such as social dominance orientation (SDO), Ambivalent Sexism, and Symbolic Racism, which encompass normative prescriptions about group relations in society independent of a particular situation (Jost 2006; Brandt 2022; Brandt et al. 2019; Brandt and Sleegers 2021). The validity of this approach can also be confirmed empirically, by testing the structure of violence beliefs under varying levels of threat (as we do in the studies described below).

Our approach underscores the significance of understanding collective violence beliefs within the broader context of intergroup relations, elucidating the intricate nature of these beliefs. While our current research focuses on developing new measures, it lays the groundwork for future studies to provide a more comprehensive insight into their antecedents and consequences through experimental and longitudinal designs. Moreover, our conceptualization mitigates social desirability concerns by concentrating on the justification of violence committed by others, enabling respondents to express their views more candidly. We believe this offers greater conceptual clarity, as it is purely attitudinal, in contrast to scales that combine attitudes and behaviors.

### 1.4 The Present Research

In this research, we develop and test a new scale of collective violence based on the principles and dimensions discussed earlier. We do so using samples from Lebanon, the Syrian diaspora, and Turkey. Sectarian violence in Lebanon is ongoing, and the large number of sects involved makes the intergroup dynamics quite complex. The civil war in Syria has led to millions of Syrians fleeing their country in fear for their lives. While Turkey enjoys a relatively more stable political system, the violent intergroup conflict between the Turks and the Kurds has been ongoing for decades. Additionally, conducting research in these contexts addresses the call to move beyond WEIRD samples (Henrich et al. 2010).

We first develop this scale in Lebanon due to ongoing intergroup sectarian violence. Lebanon was subject to a civil war in 1975–1991 largely along sectarian lines, fought between Christians, Sunni Muslims, Shia Muslims, Druze, with significant intervention from Palestinian, Israeli, and Syrian forces. While the civil war has ended and Lebanon has.a consociational government, low‐level violence still occurs (Saouli 2019). Furthermore, political divisions still largely reflect sectarian rather than ideological divides (Salloukh 2024).

In Study 1, we used two Lebanese samples: in Study 1a, we developed the scale and explored its factor structure, and in Study 1b, we validated the factor structure with a new sample and tested its construct and criterion‐related validity. In Study 1a, we explore the factor structure of a large set of items based on the original taxonomy we have developed based on prior intergroup violence literature (see Parrott and Giancola 2007), covering acts that are direct and indicative of the following subtypes: Physical, Verbal, Postural, Symbolic Violence, and Destruction of Property. In Study 1b, we further develop the scale by confirming the factor structure ascertained from Study 1a and making minor modifications to item content. In Study 2, we examine whether priming intergroup threat affects the structure of the scale. In Study 3, we replicate the threat findings from Study 2 and further confirm the factor structure of the scale outside the Lebanese context (i.e., among the Syrian Diaspora). Study 4 strengthens the ecological validity of the scale by confirming its psychometric properties in Turkey.

### 1.5 Ethical Statement

This research was conducted in strict adherence to ethical standards and received the necessary ethical approvals. We ensured the anonymity of results and upheld participant autonomy by allowing them to withdraw from the survey at any time. Additionally, all survey questions were non‐mandatory, and we provided participants with a list of psychological support organizations at the conclusion of the survey for their reference and support.

## 2 Study 1

### 2.1 Methods

The data that support the findings of this study are openly available in OSF at https://osf.io/yz5ba/?view_only=0ca9ce7e4372433daa2f248508c8de33.

### 2.2 Procedure

#### 2.2.1 Phase I: The Dimensionality of Collective Violence Beliefs

Using best practices in scale development (DeVellis and Thorpe 2021), we took several steps in developing the collective violence scale. Initially, we reviewed relevant literature in collective violence and interpersonal violence. We also reviewed available scales that have been used in interpersonal violence (for a full review see Parrott and Giancola 2007). Additionally, we reviewed theoretical frameworks that treat violence as multidimensional (e.g., Parrott and Giancola 2007; Winiewski and Bulska 2020).

After a comprehensive review, we developed 43 items for our scale, as detailed in Supporting Information Table S1. These items, inspired by the taxonomy of Parrott and Giancola (2007), were crafted following consultations done by the lead author with multiple stakeholders knowledgeable about conflict settings in Lebanon and Syria. While not encompassing every dimension of the taxonomy, our selection included all dimensions deemed relevant to intergroup violence. This relevance was determined based on our extensive observation of intergroup conflict incidents over the past century (Correlates of War, [n.d.]; International Policy Institute for Counter Terrorism, [n.d.]; Wars and Conflict, [n.d.]). In addition to the subtypes mentioned in the taxonomy, we included one additional subtype (symbolic violence) based on our observation of intergroup conflict. The original scale we used included the following subtypes: physical violence (e.g., hitting someone), verbal violence (e.g., verbally attacking someone), postural violence (e.g., threating someone with their body posture), symbolic violence (e.g., burning an effigy that represents someone), and destruction of property (e.g., destroying a property that belongs to someone with the purpose of causing harm). The developed items were translated into Arabic by two independent sworn translators and then back translated into English by a different translator. The lead researcher, a native Arabic speaker, confirmed that the meaning of the items was preserved before proceeding.

Phase I of the scale development process involved administering these 43 items to a diverse community sample in Lebanon and exploring the factor structure that emerged (Study 1a). The aim was to test which items loaded most strongly onto the underlying dimensions and select the subset of items that would produce the most reliable multi‐dimensional scale for future use in collective violence research.

#### 2.2.2 Phase II: Confirming Dimensionality and Modifying Item Set

In Phase II, we modified the item set to account for the results of Phase I. Specifically, we added 24 new items and removed the problematic items that were not retained based on their performance in Phase I. These additional items were developed in a similar manner to the first attempt, however, while acknowledging the two dimensions that emerged in the EFA of the first phase. We aimed to recreate the factor structure in Phase I, with items that better matched the dimensionality revealed in Phase I. To do so, we administered this new set of items to another large, diverse community sample in Lebanon (Study 1b).

#### 2.2.3 Phase III: Criterion Validity

In Phase III, we used data from both Study 1a and 1b to test the criterion validity of the scale – specifically, the degree to which the dimensions of our new scale correlated with existing measures of theoretically relevant constructs. Table 1 shows the validation constructs available across the two samples used in Study 1.

**Table 1:** cviolence.

		1	2
7.	It is justified for members of my sect to become physically aggressive towards members of a different sect	**0.910**	−0.079
1.	It can be justified for members of my sect to hit members of a different sect	**0.949**	−0.083
3.	It can be justified for members of my sect to physically harm members of a different sect	**0.906**	−0.144
8.	It is justified for members of my sect to verbally insult members of a different sect	**0.914**	0.082
12.	It is sometimes justified for members of my sect to verbally threaten members of a different sect	**0.838**	0.102
13.	It can be justified for members of my sect to verbally assault members of a different sect	**0.884**	0.130
36.	Destroying the property of a different sect is sometimes a justified reaction	**0.861**	−0.013
37.	It is justified sometimes for members of my sect to destroy property of members from a different sect	**0.877**	−0.087
40.	It is justified sometimes for members of my sect to damage cars of members from a different sect	**0.876**	−0.061
10.	Cursing politicians can be justified	−0.089	**0.787**
14.	Verbally insulting politicians can be justified	−0.055	**0.773**
20.	Generally, offending political leaders through drawings, falls under freedom of expression	−0.155	**0.713**
28.	Holding symbolic trials and hanging effigies representing enemy figures is a justified act	0.207	**0.704**
29.	It is accepted that people share memes making fun of politicians they do not agree with	−0.022	**0.779**
32.	Holding symbolic trials and hanging effigies representing corrupted politicians is a justified act	0.040	**0.819**
33.	Building representative figures of corrupted politicians and destroying them in groups is a justified act	0.138	**0.801**
35.	Generally, people interact positively with pictures that make fun of politicians they don't like on social media	−0.122	**0.629**

*Note:* Estimated via exploratory structural equation modeling (ESEM) framework in *lavaan*. Rotation Method: Varimax with Kaiser Normalization. All items treated as ordinal. Factor loading characteristic of specific factors are indicated in bold.

If our new scale validly captures collective violence beliefs, it should correlate positively with other aggression measures, including interpersonal aggression. Thus, we test the correlation between our dimensions and the four dimensions of the most commonly used measure of interpersonal violence: The Buss Perry Questionnaire (BPQ; Buss and Perry 1992). Our scale should also correlate positively with other measures of collective behavior typically studied in social psychology, specifically, non‐normative and violent non‐normative collective action (Tausch et al. 2011). Violence beliefs relating to outgroups should also correlate negatively with tolerance of outgroups. In Study 1a, we operationalised tolerance towards outgroups in terms of a single item feeling thermometer. In Study 1b, we used a more reliable, multi‐item measure developed by Roccas and Brewer (2002).

As a second step, we test whether other constructs (i.e., perceived efficacy, system justification, and religious fundamentalism) would be differential predictors of our dimensions. Based on prior research, we expect collective violence to correlate negatively with perceived group efficacy (Becker and Tausch 2015; Tausch et al. 2011) and system justification (D. Osborne et al. 2019), and positively with religious fundamentalism (Lobo 2009).

### 2.3 Participants

#### 2.3.1 Study 1A

An adult convenience sample was collected by circulating an anonymous Qualtrics link via social media, using the lead author's connections with six community groups in Lebanon in December 2020. The survey was completed by 1170 individuals. However, the sample used for the purpose of this study consisted of 596 participants due to missing responses on items related to collective violence beliefs. This can be justified as responses to every question were not required for survey completion, and some participants might have felt uncomfortable or unwilling to answer questions related to collective violence beliefs. The sample was broadly representative of the various sectarian groups in this diverse country (Mean Age: 28.71 [SD = 9.61], 58.5% female). Specifically, the sample composition was 17.3% Christian Maronite, 15.1% Shi'a, 32.5% Sunni, 23.4% Druze, 13.7% Christian Orthodox, 0.9% Armenian, 4.1% other Christian sects, and 3% other Muslim sects. Additionally, 21.1% of the sample identified themselves as politically affiliated with the October Revolution of 2019, while the remaining percentage were either members of traditional parties or other political groups.

#### 2.3.2 Study 1B

A second adult convenience sample was collected in April 2021 by circulating an anonymous Qualtrics link via social media, using the lead author's connections with six community groups in Lebanon. The survey was completed by 2311 individuals. However, the sample used for the purpose of this study consisted of 1035 participants (Mean Age: 31.05 [SD 18.10], 47% female) due to missing responses on certain items related to collective violence beliefs. This sample captured a similar level of diversity to the Study 1a sample. Specifically, the sample composition was 29.5% Christian Maronite, 15.9% Shi'a, 23.9% Sunni, 13.3% Druze, 9% Christian Orthodox, 0.4% Armenian, 5.6% other Christian sects, and 1.9% other Muslim sects. The majority of the sample did not identify with any political group, including alternative groups that emerged after the 2019 revolution.

### 2.4 Results

#### 2.4.1 Phase I

To identify the initial factor structure of our collective violence scale (see Supporting Information Table S1 in the Supplemental Information for the full list of items), which was translated into Arabic by independent sworn translators and back‐translation, we conducted an exploratory factor analysis (EFA) using maximum likelihood extraction and varimax rotation. Maximum likelihood extraction produced a five‐factor solution; however, the scree plot indicated a sharp “bend of the elbow” between the second and third factor mark. The initial eigenvalues indicated that the first factor accounted for 33.95% of the variance, the second factor for 12.72%, and the third factor for 4.11%. The cumulative percentage of variance explained by these three factors was 50.77%. Similarly, the extraction sums of squared loadings showed that the first factor explained 32.78% of the variance, the second factor 11.65%, and the third factor 2.70%, resulting in a cumulative percentage of 47.13%. The rotation sums of squared loadings indicated that the first factor accounted for 30.10% of the variance, the second factor for 9.80%, and the third factor for 6.69%, with a cumulative percentage of 46.56%. We proceeded to examine the factor loadings and remove problematic items (Tabachnick and Fidell 2007). Only items with primary loadings higher than 0.50 and cross‐loadings equal to or less than 0.20 were retained (Osborne and Costello 2009), and after re‐examining the fit statistics, we were left with three factors and 33 items.

After this exercise, we examined the factor loadings again, and it was evident that items did not load together based on the nature of the behavior (physical, verbal, postural, symbolic, destruction of property). Instead, items loaded together because of the target of the act. Items that were targeted against ordinary members of a different sect (outgroup) loaded on the same factor regardless of the act. These acts, which we call diffuse collective violence (acts against ordinary outgroup members), comprise Factor 1. Items that were targeted against outgroup politicians or enemy figures, which we call upward collective violence (acts against outgroup leaders or enemy figures), also loaded on a second factor regardless of the act. None of the items had loadings above 0.40 on the third factor, so we stopped adding factors and did not interpret the third factor further.

All eight items which included acts of collective violence against politicians or enemy figures loaded on the second factor. The remaining items loaded on the first factor, which resulted in the first factor holding most items. To resolve this imbalance, we decided to retain items loading on factor one higher than 0.70 and cross‐loadings equal to or less than 0.10. We note that this process was carried out treating items as continuous. However, in light of reviewer feedback that the skewed nature of variable distributions toward strong disagreement (see Supporting Information Table S10 for a breakdown of item distributions) with the items makes treating them as ordinal more appropriate, estimates of loadings in this paper are now done treating items as ordinal. We retained 17 items in total, nine loading on factor one and eight loading on factor two, and then re‐examined the fit statistics and loadings on a case‐by‐case basis.

During this process, no item loaded on the third factor, and the maximum likelihood extraction produced a two‐factor solution. The first factor explained 42.7% of variance, the second factor 27.1% (total: 69.9%). The two‐factor structure retained this diffuse versus upward distinction (see Table 1). Both diffuse collective violence and upward collective violence showed high internal reliability (ω_diffuse_ = 0.93, ω_upward_ = 0.95). The model also displayed good fit to the data (RMSEA = 0.067 [90% CI: 0.059, 0.074], SRMR = 0.037, CFI = 0.981, TLI = 0.975), and superior fit compared to a one‐factor model (RMSEA = 0.221 [90% CI: 0.215, 0.227], SRMR = 0.368, CFI = 0.761, TLI = 0.727). While switching to treating items as ordinal meant cross‐loadings greater than 0.10 existed for both factors, they were still generally small. Loadings and model fit are nearly identical when we use a rotation method (Oblimin) that allows the factors to correlate (see Supporting Information Table S15).

#### 2.4.2 Phase II

In Phase II (Study 1B), we revised our initial item set to better align with the two‐dimensional structure identified in Phase I, which differentiated between diffuse and upward collective violence beliefs. We added 24 new items to further capture the concept of upward collective violence, as revealed in Phase I, in order to cover the criterion space more comprehensively (e.g., Being verbally violent against those who got us here is a justified act; Holding symbolic trials and hanging effigies representing corrupted politicians is a justified act). We performed an exploratory factor analysis (EFA) using maximum likelihood extraction and varimax rotation on this revised item set.

The maximum likelihood extraction yielded a six‐factor solution; however, the scree plot indicated a sharp “bend of the elbow” between the fourth and fifth factor mark. Consequently, we examined the factor loadings. Our initial analysis revealed that all nine items targeting violence at outgroup members, retained from Study 1a, loaded together above 0.60, with no cross‐loadings above 0.20. After removing items with primary loadings below 0.50 and cross‐loadings equal to or higher than 0.20 (Osborne and Costello 2009), we were left with four factors and 24 items.

We repeated this process with the remaining items, which resulted in a two‐factor structure with 18 items (see Supporting Information Table S11 for distributions of item answers). Table 2 depicts factor loadings. The first factor explained 37.4% of variance, the second factor 35.3% (total: 73.0%). Consistent with our findings in Phase I, the factor structure distinguished between diffuse and upward collective violence, and the scale showed high reliability (ω_diffuse_ = 0.93, ω_upward_ = 0.94). The model displayed good fit on most indicators (RMSEA = 0.098 [90% CI: 0.091, 0.105], SRMR = 0.035, CFI = 0.964, TLI = 0.954) and superior fit to a single‐factor model (RMSEA = 0.213 [90% CI: 0.206, 0.219], SRMR = 0.408, CFI = 0.807, TLI = 0.782). Factor loadings and model fit were nearly identical when we used Oblimin rotation, which allowed factors to correlate (see Supporting Information Table S16). We named this final scale (see Supporting Information Tables SI2–SI9) the Two‐Dimensional Collective Violence Beliefs Scale (CVBS: 2D).

**Table 2:** Factor loadings of the final items included in the two‐dimension collective violence beliefs scale (CVBS: 2D).

		1	2
1.	It is justified for members of my sect to become physically aggressive towards members of a different sect	**0.894**	−0.029
2.	It can be justified for members of my sect to hit members of a different sect	**0.892**	0.068
3.	It can be justified for members of my sect to physically harm members of a different sect	**0.869**	0.058
4.	It is justified for members of my sect to verbally insult members of a different sect	**0.841**	0.058
5.	It is sometimes justified for members of my sect to verbally threaten members of a different sect	**0.837**	0.044
6.	It is sometimes justified for members of my sect to verbally insult members of a different sect on social media	**0.848**	−0.003
7.	Destroying the property of a different sect is sometimes a justified reaction	**0.733**	0.097
8.	Burning cars of members from a different sect can be a justified act	**0.828**	−0.015
9.	It is justified sometimes for members of my sect to damage cars of members from a different sect	**0.836**	0.013
10.	Verbally insulting politicians can be justified	−0.009	**0.879**
11.	One can justify people's need to be violent towards our country's leaders	0.069	**0.840**
12.	Dragging effigies representing corrupted politicians in the streets is a justified act	−0.009	**0.878**
20.	Insulting those responsible for our situation is a justified act	0.025	**0.824**
24.	Destroying property of corrupted politicians can be justified	0.070	**0.811**
27.	Holding symbolic trials and hanging effigies representing corrupted politicians is a justified act	0.002	**0.896**
29.	Being verbally violent against those who got us here is a justified act	−0.042	**0.857**
30.	Burning effigies of corrupted politicians is a justified act	−0.021	**0.937**
31.	Building representative figures of corrupted politicians and destroying them in groups is a justified act	0.079	**0.838**

*Note:* Estimated via ESEM framework in *lavaan*. Rotation Method: Varimax with Kaiser Normalization. All items treated as ordinal. Factor loading characteristic of specific factors are indicated in bold

#### 2.4.3 Phase III–Criterion Validity

Consistent with our theoretical expectations, we found positive correlations between all four dimensions of the Buss Perry Questionnaire (BPQ; Buss and Perry 1992) and our scale (see Table 3). We also discovered a positive correlation between non‐normative collective action and diffuse collective violence in Study 1a (*ρ* = 0.17, *p* < 0.01), as well as a positive correlation between violent non‐normative collective action and upward collective violence in both Study 1a (*ρ* = 0.40, *p* < 0.01) and 1b (*ρ* = 0.46, *p* < 0.01). While the correlation between non‐normative collective action and the first dimension of our scale in Study 1a is relatively weak compared to other correlations observed in our study, it still supports the criterion validity of our scale. This might indicate that the first dimension, which focuses on diffuse collective violence, is related to non‐normative collective action, though the relationship may be influenced by additional factors that warrants further exploration. Moreover, we found a negative correlation between warmth towards outgroup members and diffuse collective violence in Study 1a, and a negative correlation between tolerance towards outgroup members and diffuse collective violence in Study 1b. Surprisingly, the correlation between tolerance towards outgroup members and upward collective violence was positive.

**Table 3:** Correlations between the mean scores of DCV, UCV and various constructs (Spearman's *ρ*).

Construct	Diffuse collective violence		Upward collective violence	
	Study 1a	Study 1b	Study 1a	Study 1b
Non‐normative collective action	0.17**	0.07	0.40**	0.46**
Violent non‐normative collective action	0.28**	0.10*	0.33**	0.47**
Warmth toward outgroup	−0.09*	−0.29**	0.01	0.22**
BPQ physical aggression	0.37**	**—**	0.12**	**—**
BPQ verbal aggression	0.04	**—**	0.19**	**—**
BPQ anger	0.27**	—	0.08	**—**
BPQ hostility	0.19**	—	0.17**	**—**

* *p* < 0.05

** *p* < 0.01.

## 3 Theoretical Expectations and Ad Hoc Predictions

We also examined the relationship between perceived group efficacy, system justification, religious fundamentalism, and each of the two collective violence dimensions in our scale using SEM, while controlling for the shared covariance between our dimensions. Understanding and theorizing about the nature of these differential predictors of the two dimensions of collective violence is beyond the scope of the current paper. We did not initially expect to find the two specific dimensions revealed in Phase I and, therefore, did not make any formal predictions about differential effects across dimensions. However, based on the results, we can provide ad hoc predictions that align with social psychological theories.

### 3.1 Perceived Efficacy

Higher perceived group efficacy is expected to be associated with greater support for actions that assert group dominance and achieve ingroup goals, aligning with upward collective violence. Conversely, lower perceived group efficacy might lead to frustration and aggression directed towards outgroup members, corresponding to diffuse collective violence. We measured this using a 10‐item scale, including items such as “Most politicians are not in touch with the people” (reverse‐scored) and “I don't feel like I have a say in what the government does” (reverse‐scored) which we have adapted from various established scales (e.g., Niemi et al. 1991; Beierlein et al. 2012) on 5‐point scales from 1 (totally disagree) to 5 (totally agree) (α = 0.72).

### 3.2 System Justification

Individuals with higher system justification beliefs tend to support the status quo and existing social hierarchies. Therefore, system justification should be positively related to diffuse collective violence, as these individuals might justify aggression towards outgroup members to maintain social order. In contrast, a negative relationship with upward collective violence is anticipated, as challenging outgroup leaders would contradict their system‐supporting beliefs. We measured this construct using a 5‐item scale, including “In general, you find society to be fair” and “In general, the Lebanese political system operates as it should” adapted from Jost et al. (2004) on 5‐point scales from 1 (totally disagree) to 5 (totally agree) (α = 0.48). The scale initially had 7 items, but we omitted 2 because they led to lower scale reliability.

### 3.3 Religious Fundamentalism

Religious fundamentalism often promotes rigid adherence to traditional beliefs and opposition to social change. Consequently, religious fundamentalism should be positively related to diffuse collective violence due to its potential to foster intolerance towards outgroup members. However, a negative relationship with upward collective violence is expected, as such acts might disrupt the perceived religious and social order. We measured this using a 12‐item scale, including “God has given humanity a complete, unfailing guide to happiness and salvation, which must be totally followed” and “When you get right down to it, there are basically only two kinds of people in the world: the Righteous, who will be rewarded by God; and the rest, who will not” adapted from Altemeyer and Hunsberger (2004) on 5‐point scales from 1 (totally disagree) to 5 (totally agree) (α = 0.91).

**Figure 1:**
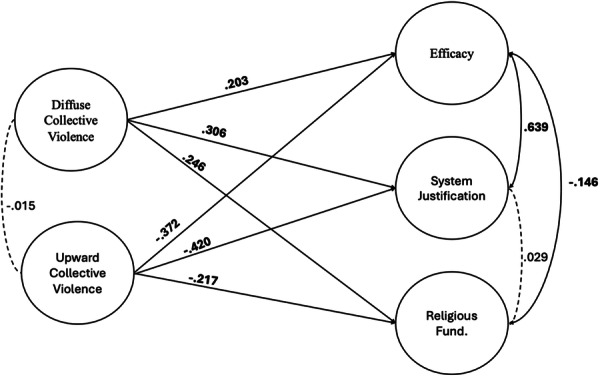
Structural equation model, Study 1B. Dashed paths = non‐significant. Statistically significant coefficients in bold.

To test these relationships, we estimated a structural equation model in which diffuse and upward collective violence predict efficacy, system justification, and religious fundamentalism. We treated all items as ordinal. Results are depicted in Figure 1. The model fit well to the data (RMSEA = 0.047 [95% CI: 0.044, 0.051], SRMR = 0.071, CFI = 0.988, TLI = 0.987).

We found, contrary to expectations, that efficacy was positively related to diffuse collective violence and negatively related to upward collective violence. In line with expectations, both system justification and religious fundamentalism were positively related to diffuse collective violence, but negatively related to upward collective violence.

These findings seem to align with social psychological theories. For instance, people who justify the system and religious fundamentalists are more favorable toward societal leaders (Lobo 2009; D. Osborne et al. 2019) and, as a result, should be more prone to expressing violence against outgroup members rather than leaders. On the other hand, those with higher efficacy may be more hesitant to potentially ruin their perceived influence by enacting violent sentiments against group leaders. Most importantly, these divergent effects underscore the importance of treating collective violence as a multi‐dimensional rather than unidimensional construct.

### 3.4 Discussion

Study 1 tested the scale for measuring support for collective violence using two samples from Lebanon. In Phase I, we developed 43 items inspired by complex multi‐dimensional taxonomies found in the interpersonal violence literature. Our analysis and repeated EFAs confirmed the multi‐dimensional nature of collective violence. However, the factor structure did not align with our initial expectations. Items of the scale loaded on two different factors not related to the intensity of the act, but to the target of said acts. Items targeted at outgroup members loaded together, while items targeted at outgroup leaders loaded together. At the end of the analysis, we retained the 17 best performing items, matching the developed theoretical assumption. Nine items loaded on support for collective violence acts against outgroup members (Diffuse Collective Violence), and eight items loaded on support for collective violence acts against outgroup leaders (Upward Collective Violence). Although our initial expectations were not met, the new factor structure aligns with theoretical reasoning. For example, following the US invasion of Afghanistan after 9/11, numerous protests occurred in various Islamic countries, where protesters burned US flags and effigies of presidents, yet American citizens were still welcome in these countries (Göttke 2020). This anecdotal evidence is empirically supported by our study. Furthermore, research on stalking has shown that politicians are more likely to be victims of violent attacks than others (Meloy 2007; Meloy et al. 2001), reinforcing the qualitative differences we found between outgroup members and leaders.

In Phase II, we tested our newly developed model by including 24 additional items to the scale we retained from Phase I. In line with our new approach, we conducted EFAs that confirmed what we found in Phase I. Items loaded on two factors only, and the factors did not differ by the intensity of the act; instead, the difference was the target group of those acts. We retained 18 items, nine on each of the two factors. We then tested for criterion validity and found that our two dimensions positively correlated with the four dimensions of the Buss‐Perry Questionnaire (BPQ) and non‐normative and violent non‐normative collective action. Warmth and tolerance towards the outgroup, as expected, correlated negatively with justification of collective violence beliefs against outgroup members but not outgroup leaders. We also tested the relationship between three known predictors of collective behavior and our scale's two dimensions: perceived group efficacy, system justification, and religious fundamentalism. We discovered that all three predicted our two dimensions differentially, further confirming the importance of including both dimensions in research on collective violence.

However, this scale has several limitations that we sought to address in the following studies. First, it was only tested in a single country and intergroup context—that is, Lebanon. Therefore, we need to know if the two‐dimensional structure can be replicated in other contexts. Additionally, the use of convenience sampling in this study limits the representativeness of the sample. While convenience sampling may not fully capture the diversity of the population, the sample included participants from various sectarian and political backgrounds, providing a diverse range of perspectives. Despite these limitations, the consistent internal reliability and the theoretically meaningful correlations observed in our data suggest that the measure holds some extent of external validity. To address these limitations nevertheless, we aim to replicate these findings. Secondly, many other violence scales treat threat as a prerequisite for violence and assess support for collective violence in reaction to specific threats (e.g., reactions to newcomers who threaten the homogeneity and lifestyle of locals, Winiewski and Bulska 2020). Our conceptualization and measurement of collective violence beliefs treat the acceptability of collective violence as something members of the population vary on, regardless of any specific threat being primed. We test this assumption empirically by ascertaining if the psychometric properties of our scale depend on the degree to which participants are made to feel threatened by a specific outgroup.

## 4 Study 2

Study 2 had four aims. First, we aimed to confirm the factor structure of the violence scale presented in the previous study. Second, we sought to explicitly clarify that the leaders in question were outgroup leaders. This clarification was important not only to ensure consistency in the interpretation of the scale items but also to enhance the scale's generalizability across different contexts. Third, we evaluated whether priming threat has a significant impact on the scale's structure. This was to demonstrate that collective violence beliefs are not just a response to salient societal threats, but are an integral aspect of intergroup psychology, in that that people vary in how much they consider violence a legitimate means to serve their ingroup's interests. Lastly, we aimed to test the possibility of creating a shorter version of the scale to enhance its usability. To this end, we retained the six best‐performing items from each subscale, resulting in a more concise scale for future applications.

### 4.1 Participants

An adult convenience sample was collected by circulating an anonymous Qualtrics link via social media, using the lead author's connections with six community groups in Lebanon. Originally, 1710 participants completed the survey. We did our best to communicate through the community organizations that the survey included items on violence and emphasized the importance of responding to these items. Although participation was voluntary and we did not mandate responses, we observed an improved response rate for the violence items compared to Study 1. The final sample consisted of 1041 participants (Mean Age: 33.07 [SD = 11.43], 65.5% female) and was broadly representative of the various sectarian groups in this diverse country (11.3% Christian Maronite, 17.5% Shi'a, 38.5% Sunni, 6% Druze, 4.4% Christian Orthodox, 0.5% Armenian, 3% Other Christian sects, 5% Other Muslim sects, 13.8% No sectarian identification). A plurality (41.1%) had no political affiliation, while the remainder varied among all the other political groups.

### 4.2 Procedure

To investigate the impact of threat on the scale's structure, participants were randomly assigned to one of three conditions.

#### 4.2.1 Subtle Threat Prime Condition (N = 341)

In this condition, we aimed to subtly prime intergroup threat among participants while they considered the items on our scale. As a result, the scale developed in Study 1 was presented with a minor modification. Each item included the qualification that the violent act is justifiable “if you feel your sect is threatened.” For instance, Item 1 on the diffuse scale dimension was presented as “It is justified for members of my sect to become physically aggressive towards members of a different sect if I feel my sect is threatened [emphasis added].”

#### 4.2.2 Strong Threat Prime Condition (N = 333)

Prior to answering the 12 items on the scale, participants were prompted to think about the sectarian conflict in Lebanon over the past 50 years and its effects on them. They were then asked to write up to three sentences explaining their feelings on the matter. Subsequently, participants completed the same scale as in Study Unlike the subtle threat prime condition, we did not include “if you feel your sect is threatened” in the items.

#### 4.2.3 Control Condition (N = 367)

In this condition, participants were presented with the original version of the scale without any additional threat primes.

### 4.3 Results

To test measurement invariance, we conducted a stepwise analysis. In the first step, we compared the two versions of the scale that were most similar—the control condition and the strong threat prime condition. These two conditions used identical items, but the latter condition primed threat via an induction task. In the next step, we tested the original scale against the subtle threat prime, in which the items were slightly different (with the latter including an additional clause that said “if I feel my sect is threatened”).

We conducted a multigroup CFA using the *lavaan* package in RStudio 12.1. To test measurement invariance, we first estimated a configural model where no constraints were imposed, followed by a metric model, in which factor loadings were constrained to be equal across the two conditions. Lastly, we estimated a scalar model, which constrained both factor loadings and thresholds to be equal across conditions (Meredith, 1993). Fit measures are depicted in Table 4, and factor loadings are depicted in Table 5. Because the distribution of items was scaled towards disagreement, particularly for diffuse collective violence, we treated items as ordinal (see Supporting Information Table S12 for distributions of answers to each item in each condition).

**Table 4:** Measurement invariance analyzes, Study 2. Bold indicates best‐fitting on that particular fit index.

	χ2	df	*p*	RMSEA	SRMR	CFI	TLI	ΔRMSEA	ΔSRMR	ΔCFI	ΔTLI
Configural	**368.154**	159	< 0.001	0.065	**0.060**	**0.987**	0.983				
Metric	389.020	179	< 0.001	0.062	0.063	**0.987**	0.985	−0.004	0.003	0.000	0.002
Scalar	484.330	247	< 0.001	**0.056**	**0.060**	0.985	**0.988**	−0.006	−0.002	−0.002	0.003

**Table 5:** Factor loadings by experimental condition, configural model, Study 2.

Factor	Wording	Loading, control	Loading, subtle threat	Loading, strong threat
Diffuse	It is justified for members of my sect to become physically aggressive towards members of a different sect	0.826	0.831	0.912
Diffuse	It can be justified for members of my sect to hit members of a different sect	0.888	0.874	0.907
Diffuse	It can be justified for members of my sect to physically harm members of a different sect	0.871	0.907	0.924
Diffuse	It is justified for members of my sect to verbally insult members of a different sect	0.861	0.822	0.836
Diffuse	It is sometimes justified for members of my sect to verbally threaten members of a different sect	0.858	0.806	0.820
Diffuse	It is sometimes justified for members of my sect to verbally insult members of a different sect on social media	0.840	0.831	00.808
Upward	Verbally insulting politicians from other sects can be justified	0.840	0.680	0.685
Upward	One can justify people's need to be violent towards our country's leaders especially those from other sects	0.800	0.724	0.636
Upward	Dragging effigies representing corrupted politicians from other sects in the streets is a justified act	0.892	0.832	0.730
Upward	Holding symbolic trials and hanging effigies representing corrupted politicians from other sects is a justified act	0.853	0.794	0.763
Upward	Burning effigies of corrupted politicians from other sects is a justified act	0.865	0.829	0.828
Upward	Building representative figures of corrupted politicians from other sects and destroying them in groups is a justified act	0.853	0.829	0.831

A χ2 test found that the scale did not achieve metric invariance (*p* = 0.004), though a model assuming scalar invariance did not reduce fit further (*p* = 0.257). That said, while fit on some indicators suffered with stricter fit assumptions (SRMR, CFI), it improved with others (RMSEA, TLI). LaGrange Multiplier Tests indicated that three items in particular failed invariance. The loading for the “physical harm” item in the Diffuse factor was significantly stronger in the strong threat prime condition than in the control condition (*p* = 0.028). The loadings for the “verbally threaten” item from the Diffuse factor and the “burning effigies” and “destroying representative figures in groups” items from the Upward factor were stronger in the control condition (p*s* < 0.020). However, loadings were high across conditions, and indicators of model fit indicated good fit across conditions.

We also conduct an Average Variance Extracted (AVE) test by condition. Factors with AVEs ≥ 0.50 tend to explain variance in their indicators well, and AVEs that exceed inter‐factor correlations indicate that the use of more than one dimension is valid (Fornell and Larcker 1981). AVEs exceeded the fit cutoff in each condition for both diffuse (Control: 0.755, Subtle Threat: 0.755, Strong Threat: 0.735) and upward factors (Control: 0.724, Subtle Threat: 0.561, Strong Threat: 0.614). The latent correlation between the diffuse and upward factors was strongest in the control condition (*ρ* = 0.674), followed by the subtle threat condition (*ρ* = 0.376), strong threat condition (*ρ* = 0.331, p*s* < 0.001). In each case, the inter‐factor correlation was lower than each factor's AVE, validating the use of of two dimensions.

### 4.4 Discussion

In Study 2, we further tested our scale, including the original developed version and two variations. While our scale does not achieve traditional measurement invariance, there is little evidence that the properties of the scale meaningfully change in response to priming threat or explicitly mentioning threat. As a result, we can conclude that the presence of threat is not a prerequisite for measuring individuals' support for collective violence.

These findings support our argument that collective violence beliefs are an integral aspect of intergroup relations and are not solely primed by threat. They also suggest that, at least in conflict‐prone contexts such as Lebanon, that while threat can mildly alter some scale loadings, attitudes toward collective violence retain their dimensionality. This is in contrast to research by Winiewski and Bulska (2020), who did find priming threat changed the dimensionality of attitudes toward collective violence.

## 5 Study 3

Study 3 replicated Study 2's experiment, testing the collective violence scale among Syrians outside Syria, who fled during the Syrian Civil War. The conflict existed along multiple fronts: an Alawite‐dominated government, an opposition consisting of some pro‐democracy and Islamist groups that were eventually backed by Turkey, the Kurdish‐dominated Syrian Democratic Forces, and jihadist groups. The difficulties of surveying the population in an active civil war precluded surveying the entire population. Therefore, this study focusses on a convenience sample of the opposition, and scales employed treat the conflict as regime vs opposition.

### 5.1 Participants

The survey was originally completed by 612 participants, and the responses used in this analysis are from those who provided enough answers for our analysis to run. The Syrian opposition diaspora members ranged across multiple countries, primarily in the EU and Turkey. A convenience sample of 521 adult participants (Mean Age: 39.65 [SD = 13.36], 42.7% female) was collected via a Qualtrics link shared on social media, using the lead author's connections with Syrian opposition community members in the diaspora.

### 5.2 Scale Development

Scales from the previous study were adapted to the Syrian conflict context. Specifically, the scale asked participants about outgroup members who were specified as regime supporters for the first dimension (diffuse collective violence) and regime politicians for the second (upward collective violence). The study had three conditions like Study 2 (N_control_ = 178, N_strongthreat_ = 189, N_subtlethreat_ = 154). Because the distribution of items was scaled toward disagreement, particularly for diffuse collective violence, we treated items as ordinal (see Supporting Information Table S13 for distributions of answers to each item in each condition).

### 5.3 Results

Just as with Study 2, we estimated multigroup CFAs using *lavaan* in RStudio, comparing configural models with models assuming metric and scalar invariance. Fit indices are depicted in Table 6, while factor loadings are depicted in Table 7. In contrast to Study 2, we found that the model achieved metric invariance (*p* = 0.085) and scalar invariance (*p* = 0.967). Fit was generally similar across models regardless of assumptions imposed. Strong correlations existed between the diffuse and upward factors across conditions (control: *ρ* = 0.638; Subtle Threat Prime: *ρ* = 0.777; Strong Threat Prime: *ρ* = 0.780, p*s* < 0.001). The 12 items had strong loadings, and AVEs indicate both the diffuse (AVE_control_ = 0.845, AVE_subtle_ = 0.837, AVE_strong_ = 0.845) and upward (AVE_control_ = 0.840, AVE_subtle_ = 0.869, AVE_strong_ = 0.876) scales explain a high amount of variance in their indicators. The AVEs also exceed correlations between the dimensions, validating the use of two dimensions.

**Table 6:** Measurement invariance analyzes, Study 3. Bold indicates best‐fitting on that particular fit index.

	χ2	df	*p*	RMSEA	SRMR	CFI	TLI	ΔRMSEA	ΔSRMR	ΔCFI	ΔTLI
Configural	**262.230**	159	< 0.001	0.063	**0.030**	**0.997**	0.996				
Metric	287.791	179	< 0.001	0.061	0.032	**0.997**	**0.997**	−0.002	0.003	0.000	0.000
Scalar	386.396	247	< 0.001	**0.059**	**0.030**	0.996	**0.997**	−0.002	−0.003	−0.001	0.000

**Table 7:** Factor loadings by experimental condition, configural model, Study 3.

Factor	Wording	Loading, control	Loading, subtle threat	Loading, strong threat
Diffuse	It is justified for the opposition to become physically aggressive towards regime supporters	0.920	0.943	0.972
Diffuse	It can be justified for the opposition to hit regime supporters	0.923	0.909	0.910
Diffuse	It can be justified for the opposition to physically harm regime supporters	0.932	0.918	0.943
Diffuse	It is justified for the opposition to verbally insult regime supporters	0.930	0.903	0.911
Diffuse	It is sometimes justified for the opposition to verbally threaten regime supporters	0.890	0.901	0.871
Diffuse	It is sometimes justified for the opposition to verbally insult regime supporters on social media	0.921	0.914	0.905
Upward	Verbally insulting regime politicians can be justified	0.872	0.904	0.883
Upward	One can justify people's need to be violent towards our country's leaders especially those from the regime	0.894	0.924	0.881
Upward	Dragging effigies representing corrupted regime politicians in the streets is a justified act	0.961	.937	0.927
Upward	Holding symbolic trials and hanging effigies representing corrupted regime politicians is a justified act	0.950	0.917	0.940
Upward	Burning effigies of corrupted regime politicians is a justified act	0.967	0.961	0.926
Upward	Building representative figures of corrupted regime politicians and destroying them in groups is a justified act	0.967	0.948	0.938

### 5.4 Discussion

In Study 3, we replicated the methodology of Study 2 in a different context, specifically among the Syrian diaspora. We adapted the scales and their variations to suit the current Syrian context from the opposition's perspective. Measurement invariance analysis confirmed that all three variations of the scale demonstrated a good fit, with negligible differences between the conditions. This study validated the CVBS: 2D in a second intergroup context, and reinforced the notion that priming threat is not a prerequisite for assessing variations in collective violence beliefs.

## 6 Study 4

In the final study, we aimed to confirm the factor structure of our scale in another context, specifically Turkey. In this setting, Turks constitute the advantaged ruling majority, while Kurds represent the largest disadvantaged minority (Çoksan and Cingöz‐Ulu 2022). The two groups have a long history of conflict, with Kurds experiencing discrimination in Turkey (Uluğ and Cohrs 2016). For example, majority‐Kurdish regions receive disproportionately less governmental investment, such as economic resources and educational opportunities, compared to majority‐Turk regions (Kirisci and Winrow 1997). Additionally, Kurds are not entitled to government services in their native language. The low trust between Turks and Kurds (Çelebi et al. 2014) and the presence of reciprocal negative stereotypes (Bilali et al. 2014) indicate a conflictual context where our scale should be applicable.

### 6.1 Participants and Procedure

We collected an adult convenience sample by distributing an anonymous Qualtrics link via email, leveraging some of the authors' connections within the Turkish community. The sample consisted of 194 Turkish participants (Mean Age: 24.68 [SD = 7.45], 72.8% female). The majority of the sample was collected from Turkish students in Turkish universities. All 194 participants, who were Turks, answered our questions. All participants were specifically selected to be Turks, as we were interested in examining their relationship with Kurds, a different ethnic group in Turkey. Participants answered the same items as in previous studies but adapted to the context, using the original 18 items (9 for DCV and 9 for UCV) retained from Study 1 (e.g., “It is justified for Turks to be physically aggressive towards Kurds,” “Verbally insulting some Kurdish politicians can be justified”). All items were translated into Turkish.

### 6.2 Results

We conducted a CFA to confirm the factor structure of the scale, consisting of collective violence towards outgroup members and collective violence towards outgroup leaders. Factor loadings are depicted in Table 8. Due to strong skewing of answers toward disagreement (see Supporting Information Table S14 for distributions of answers to each item), we treated all items as ordinal. The two‐factor model fit well on most indicators (RMSEA = 0.094 [90% CI: 0.083, 0.106], SRMR = 0.071, CFI = 0.991, TLI = 0.990). The two factors were strongly correlated (*ρ* = 0.564, *p* < 0.001), but fit was still better with a 2‐factor solution than a single‐factor solution (RMSEA = 0.179 [90% CI: 0.169, 0.190], SRMR = 0.179, CFI = 0.967, TLI = 0.963).

**Table 8:** Factor loadings of the final items included in the two‐dimension collective violence beliefs scale (CVBS: 2D), Study 4 (Continuous Measures).

		1	2
1.	It is justified for Turks to be physically aggressive towards Kurds.	**0.853**	
2.	It may be justified for Turks to hit Kurds.	**0.943**	
3.	It may be justified for Turks to physically harm Kurds.	**0.919**	
4.	It may be justified for Turks to verbally insult Kurds.	**0.944**	
5.	It may sometimes be justified for Turks to verbally threaten Kurds.	**0.869**	
6.	It may sometimes be justified for Turks to verbally insult Kurds on social media.	**0.891**	
7.	Destroying the properties of Kurds is sometimes a justified reaction.	**0.956**	
8.	Burning the cars of Kurds may be a justifiable action.	**0.975**	
9.	It is sometimes justified for Turks to damage the cars of Kurds.	**0.977**	
10.	Verbally insulting some Kurdish politicians can be justified.		**0.796**
11.	One can justify people's need to be violent towards some Kurdish politicians		**0.853**
12.	Insulting some Kurdish politicians who are responsible for our situation is a justified act		**0.728**
13.	Dragging effigies representing some corrupted Kurdish politicians in the streets is a justified act		**0.924**
14.	Destroying properties of some corrupted Kurdish politicians can be justified		**0.873**
15.	Holding symbolic trials and hanging effigies representing some corrupted Kurdish politicians is a justified act		**0.924**
16.	Being verbally violent against some Kurdish politicians who got us here is a justified act		**0.767**
17.	Burning effigies of some corrupted Kurdish politicians is a justified act		**0.975**
18.	Building representative figures of some corrupted Kurdish politicians and destroying them in groups is a justified act		**0.983**

*Note:* Estimated using *lavaan* in R. Loadings reflect those after modification indices. Factor loading characteristic of specific factors are indicated in bold.

As part of the refinement process, we found allowing errors to correlate between three sets of items would improve model fit: the “hit” and “physically harm” Diffuse items, the “verbally threaten” and “verbally insult on social media” Diffuse items, and the “Insulting” and “Being verbally violent” Upward items. When we allow these errors to correlate, model fit improves (RMSEA = 0.082 [90% CI: 0.069, 0.094], SRMR = 0.071, CFI = 0.993, TLI = 0.992). The AVEs for each factor exceed 0.50 (Diffuse: 0.858, Upward: 0.763), and exceed the inter‐factor correlation, showing good fit to the indicators and validating the use of two dimensions.

### 6.3 Discussion

In the final study, we tested our scale in a different context—Turkey. While it was clear that our two‐factor solution produced better fit than a single factor containing all items tapping attitudes toward collective violence, fit was not as good as in other contexts. We believe this is possibly due to small sample size. Strong factor loadings suggest that the scale's underlying structure remains robust.

## 7 General Discussion

In five samples covering sectarian conflict (Lebanon), a civil war fought along regime‐opposition and sectarian lines (Syria), and ongoing secessionist ethnic conflict (Turkey), we conceptualized and developed a two‐dimensional measurement scale for collective violence beliefs. Study 1 validated the scale using two samples from Lebanon, demonstrating the multidimensionality based on the target of the act rather than its nature or intensity. Studies 2 (Lebanon) and 3 (Syrian diaspora) confirmed the factor structure, showing it was consistent regardless of whether threat was primed. Study 4 further confirmed the scale's factor structure in the Turkish context (See Supporting Information Tables S2–S9 for scale items in English, Arabic, and Turkish).

Our Collective Violence Beliefs Scale (CVBS: 2D) encompasses two dimensions: Diffuse Collective Violence (DCV), targeting outgroup members, and Upward Collective Violence (UCV), targeting outgroup leaders. Both dimensions include items of varying intensity and nature. The differing mean scores for DCV and UCV among the samples demonstrate the scale's sensitivity to diverse conflict dynamics, capturing perceptions in a multidimensional manner. This is crucial, given that different predictors have varying effects on support for distinct types of collective violence. Furthermore, despite correlating strongly in a number of studies, both DCV and UCV factors are required to achieve good fit to the data, and both have different correlates and consequences.

We note that in Studies 1a and 1b, the two dimensions did not correlate at all, while they often did strongly in Studies 2–4. This pattern is unlikely to index country‐level differences, as Study 2, just like Studies 1a and 1b, took place in Lebanon. This pattern is also not likely to be attributable to scale type. Studies 1a and 1b used a larger set of items, but so did Study 4. Instead, the circumstances of Studies 1a and 1b were likely unusual. Both studies were collected close to the start of the 2019 Lebanese Revolution, a time where Lebanese citizens may have particularly motivated to distinguish between outgroup members and outgroup leaders, as the latter were often blamed for deteriorating economic and security conditions. By contrast, Study 2 took place several months after its start, and Studies 3 and 4 took place in contexts of longstanding conflict. While future work would do well to disentangle this further, these findings suggest that while distinct dimensions, DCV and UCV may come to increasingly inform one another as conflicts go on.

The three conditions used in Studies 2 and 3 revealed that introducing threat through past or present intergroup conflicts does not fundamentally alter how people perceive collective violence. While the subtle threat prime increased mean support for each type of violence, the dimensionality of support remained consistent, with differentiation between outgroup members and leaders regardless of act nature. Methodologically, this indicates our scale does not need to prime threat in order to be used. Substantively speaking, it could indicate, in line with Social Identity Theory, that threat is already embedded in intergroup relations (Tajfel et al. 1979). Even in the absence of the immediate risk of physical harm, the presence of intergroup tension and division enables individuals to think of outgroups as potential threats and endorse some level of violence against members and leaders accordingly. However, given that all of our samples are in (post‐)conflict contexts where conflict may be of heightened salience, this statement should be taken with caution.

Our scale is among the first to measure collective violence beliefs, and its development highlights three critical implications for research on collective violence. First, the scale demonstrates the multidimensionality of collective violence support, similar to interpersonal violence (Parrott and Giancola 2007). Researchers should treat the phenomenon accordingly, as different dimensions have differential predictors and consequences. Future research should focus on determining the unique predictors and outcomes of the dimensions revealed in this study.

Second, the scale confirms that its multidimensionality is based on the target group, rather than the act's intensity or nature. Not finding dimensions based on intensities suggests that people, at least in contexts of conflict, do not differentiate between acts of collective violence based on their severity. Instead, they view different tools of collective violence towards a given target as globally acceptable or unacceptable. The implications of this finding include refining our understanding of collective violence and its causes and reconsidering how we approach the phenomenon in future research.

Although our study identified two dimensions, there remains an avenue for future research to explore other potential dimensions (for instance, ingroup members vs. ingroup leaders). Another pivotal empirical question to be examined is whether in societies with multiple social groups, the explicit designation of the outgroup influences individuals' justification for violence. Despite our findings not suggesting any changes in the factor structure of our scale as a result of this, it remains an important question to further delve into. Thus, while our scale has laid substantial groundwork, continued exploration of other dimensions could yield even more comprehensive results.

Third, the measurement invariance analysis suggests that support for collective violence is not solely a reactive response to threat. Justification for violence may also be proactive, forming part of an ideological system endorsing violence as a political strategy for advancing ingroup goals. The proactive use of violence to advance ingroup goals can be understood within several theoretical frameworks. According to social identity theory (Tajfel et al. 1979), individuals derive part of their identity from their group memberships, leading to a desire to maintain a positive social identity by enhancing their group's status. In contexts where intergroup competition is high, this can manifest as support for violence to assert dominance and achieve ingroup goals. Similarly, social dominance theory (Sidanius and Pratto 1999) suggests that individuals in dominant groups may support violence to maintain their group's superiority and hierarchical position. Group deprivation theory further posits that perceived injustices and deprivations faced by the ingroup can motivate members to endorse violence as a means of redressing grievances and achieving social justice. This also aligns with research on interpersonal violence, which has shifted from viewing violence as purely reactive to considering instrumental violence for specific ends (Allen and Anderson 2017; Anderson and Bushman 2002). Importantly, this means researchers can use our scale without priming threat.

The study and scale also highlight the distinctiveness of beliefs related to outgroup leaders, an area that is often under‐explored in intergroup attitudes literature. As targets of political acts, outgroup leaders occupy a unique position (Meloy 2007). While expressing frustration against outgroup leaders can be perceived by some as an act related to the right of expression, we argue that this does not alter the nature of the acts themselves. Dragging an effigy or engaging in verbal aggression against a politician may fall within the realm of freedom of speech, particularly in WEIRD countries where such expressions are often tolerated. However, this does not mean that these acts do not hold justifications for violence. In fact, the increase in violent behavior in countries that emphasize freedom of expression, such as the United States, serves as evidence that supports our argument, especially when considering acts that we classify as collective violence in this context. Recognizing people's attitudes towards group leaders, particularly violent attitudes directed against them, is essential for understanding the dynamics of intergroup conflict and how these attitudes may manifest in various forms of collective violence.

Furthermore, the diligent methodology employed in developing our scale and our findings indicating that the perception of collective violence is determined more by the target than by the nature of the act, underscore the fact that context plays a crucial role. Consequently, we posit that our scale offers not just quantitative measurement, but also qualitative insights. Our discovery that people do not differentiate between the nature of the acts provides a valuable guide for researchers, enabling them to incorporate or disregard acts they deem more or less pertinent to their specific context.

For instance, in less confrontational societies, the inclusion of additional items focusing on verbal violence might hold greater contextual relevance, while still preserving the fundamental principle underpinning the development of this scale: The perception of collective violence is primarily driven by the target group, rather than the intensity of the violence. Thus, our scale offers a nuanced tool that can be tailored to the context under study, enhancing the depth and applicability of the research. However, it is important to note that any adaptations of the scale must undergo psychometric testing to ensure that the modified version retains its validity and reliability in the new context.

While our study employed convenience samples, we acknowledge that these samples may not fully represent the broader populations in terms of socio‐economic characteristics such as education, economic class, and age. Our samples, recruited through social media and community groups, may overrepresent individuals with higher education levels or those more engaged in social issues. This limitation might affect the generalizability of our findings. However, the diverse sectarian backgrounds of our participants, especially in Lebanon ‐ where sectarian and identity politics are very important predictors of political and social dynamics ‐ provide valuable insights into the dynamics of collective violence across different intergroup contexts. Future research should aim to validate our scale in more representative samples, including varied socio‐economic backgrounds, to ensure broader applicability, and potentially uncover other interesting patterns. Additionally, further studies should explore how socio‐economic factors may influence the perception and justification of collective violence, enhancing the scale's usability in diverse populations.

Another limitation of this paper is that our scale's properties were not tested in a WEIRD context. However, we see no reason to argue that the scale would function differently in such contexts. We encourage researchers to extend our scale to WEIRD contexts, where both diffuse and upward violence have become increasingly salient. The scale may also provide valuable insights into understanding individual differences in collective violence beliefs in historically non‐conflictual contexts that are becoming more contentious. Another limitation is the lack of exploration of potential contextual differences affecting the scale's performance. While validated across various contexts, the scale's applicability may differ in other settings due to cultural factors or conflict dynamics. Additionally, potential limitations related to methodology or sample size could affect the generalizability of our findings.

A final methodological limitation is that in many contexts, responses to the scale are skewed towards strong disagreement. When dealing with skewed distributions, we recommend that analysts specify models treating items as ordinal, whether in a structural equation modeling or ordered logit regression framework, or even dichotomize items if distributions are sufficiently skewed. Such a methodological decision reduces the potentially strong assumption that differences in support between each scale point represent the same change in latent attitudes toward collective violence. Indeed, future work can illustrate whether the move from categorical to conditional disagreement is more consequential than other movements along the scale.

By addressing these points and further investigating the implications of not finding dimensions based on intensities, we can refine our understanding of collective violence and improve the scale's applicability in diverse contexts. Future research can focus on understanding how specific cultural, historical, or contextual factors may influence the perception of intensity or nature of collective violence and how this might affect the scale's performance.

## 8 Conclusion

Our Collective Violence Beliefs Scale (CVBS: 2D) proficiently encapsulates the multifaceted nature of collective violence beliefs across diverse intergroup contexts. It establishes that the target of violent acts—be it outgroup members (Diffuse Collective Violence) or outgroup leaders (Upward Collective Violence)—serves as the basis for the scale's multidimensionality, rather than the nature of the acts themselves. The scale exhibits both theoretical and empirical significance, establishing its criterion validity through its meaningful correlations with multiple constructs. Further, the distinct influence of various predictors on the two dimensions accentuates the qualitative and psychological differences encapsulated within the proposed multidimensionality. Notably, our findings contest the conventional view that threat is an obligatory precursor for justifying collective violence, particularly within environments marked by intergroup conflict or political turbulence.

While cognizant of its limitations and the potential for contextual variations, our scale provides significant insights into the intricate phenomenon of collective violence beliefs. As such, it can guide the development of interventions aimed at mitigating intergroup conflicts, thus contributing substantially to the discourse on collective violence and conflict resolution.

## Conflicts of Interest

The authors declare no conflicts of interest.

## Supporting information

Supplemental Information October 2025 R&R 9.

## Data Availability

The data that supports the findings of this study are openly available on the Open Science Framework (OSF) at: https://osf.io/yz5ba/overview?view_only=0ca9ce7e4372433daa2f248508c8de33. All materials and analysis scripts are available in the same repository.
