# Supplementary material for: Conceptualizing and Measuring Support for Collective Violence
Source: Aggress Behav. 2026 Feb 22;52(2):e70063. doi: 10.1002/ab.70063 (PMC12925330; doi:10.1002/ab.70063)
Supplement: Supplementary file 1 — Supplemental Information October 2025 R&R 9. [file AB-52-e70063-s001.docx]

**Supplemental Information**

**Item Wordings**

**Table SI1**: Original 43 items including all Collective Violence sub-scale

| Item |  |
| --- | --- |
| 1 | It is justified for members of my sect to become physically aggressive towards members of a different sect |
| 2 | It can be justified for members of my sect to hit members of a different sect |
| 3 | Physical aggression against certain groups can be legitimate |
| 4 | It can be justified for members of my sect to physically harm members of a different sect |
| 5 | Sometimes it is justified for members of my sect to physically attack members of a different sect |
| 6 | Physical aggression is justified if carried out on behalf of the state |
| 7 | It can be justified for members of my sect to physically assault members of a different sect |
| 8 | Sometimes it is justified for members of my sect to use physical violence against members of a different sect |
| 9 | It is justified for members of my sect to verbally insult members of a different sect |
| 10 | Sometimes it is justified for members of my sect to curse members of a different sect |
| 11 | Cursing politicians can be justified |
| 12 | Speaking wrongfully of members of a different sect on social media can be justified sometimes |
| 13 | It is sometimes justified for members of my sect to verbally threaten members of a different sect |
| 14 | It is justified for members of my sect to spread rumours about members of a different sect |
| 15 | Verbally insulting politicians can be justified |
| 16 | It is sometimes justified for members of my sect to verbally insult members of a different sect on social media |
| 17 | It can be justified for members of my sect to verbally assault members of a different sect |
| 18 | It is acceptable for people to share posts that contain verbal aggression against other groups on social media |
| 19 | Members of my sect and during an argument with a different sect may pose in an offensive way as if wishing to start a fight |
| 20 | During an argument, members of my sect can sometimes be justified to stand in an offensive pose toward members of a different sect |
| 21 | When encountering a member of an enemy state, it is justified to want to invade their space with your body as a form of threat |
| 22 | I think that it can be justified for members of my sect to threaten a different sect member with one’s facial expressions |
| 23 | It is justified for someone to threaten an offender from a different sect with their physical posture |
| 24 | Squeezing one’s teeth in front of a members from a different sect as if threatening them is sometimes a justified reaction |
| 25 | It is sometimes justified to make threatening fists against members of a different sect |
| 26 | Generally, burning flags of enemy states is a justified reaction |
| 27 | Building figures representing members of an enemy state and destroying them is a justified act |
| 28 | Generally, offending political leaders through drawings, falls under freedom of expression |
| 29 | Holding symbolic trials and hanging effigies representing enemy figures is a justified act |
| 30 | It is accepted that people share memes making fun of politicians they do not agree with |
| 31 | Symbolic burying of enemy figures is a justified act |
| 32 | Holding symbolic trials and hanging effigies representing corrupted politicians is a justified act |
| 33 | Building representative figures of corrupted politicians and destroying them in groups is a justified act |
| 34 | Replacing the head of an effigy representing the enemy with an animal head effigy is a normal expression of dissatisfaction |
| 35 | Generally, people interact positively with pictures that make fun of politicians they don’t like on social media |
| 36 | Destroying the property of a different sect is sometimes a justified reaction |
| 37 | It is justified sometimes for members of my sect to destroy property of members from a different sect |
| 38 | Burning cars of members from a different sect can be a justified act |
| 39 | Destroying private property belonging to members from a different sect can sometimes be justified |
| 40 | It is justified sometimes for members of my sect to damage cars of members from a different sect |
| 41 | Attacking the embassy of another state to get back at that state can sometimes be justified |
| 42 | Destroying enemy properties is a symbolic victory over the enemy |
| 43 | Destroying public property to get back at the government can sometimes be justified |

**Table SI2**: Final Items for Diffuse Collective Violence sub-scale

| Item |  |
| --- | --- |
| DCV1 | It is justified for members of my ingroup to become physically aggressive towards members of an outgroup |
| DCV2 | It can be justified for members of my ingroup to hit members of an outgroup |
| DCV3 | It can be justified for members of my ingroup to physically harm members of an outgroup |
| DCV4 | It is justified for members of my ingroup to verbally insult members of an outgroup |
| DCV5 | It is sometimes justified for members of my ingroup to verbally threaten members of an outgroup |
| DCV6 | It is sometimes justified for members of my ingroup to verbally insult members of an outgroup on social media |

**Table SI3**: Final Items for Upward Collective Violence sub-scale

| Item |  |
| --- | --- |
| UCV1 | Verbally insulting politicians from other groups can be justified |
| UCV2 | One can justify people’s need to be violent towards our country’s leaders especially those from other groups |
| UCV3 | Dragging effigies representing corrupted politicians from other sects in the streets is a justified act |
| UCV4 | Holding symbolic trials and hanging effigies representing corrupted politicians from other groups is a justified act |
| UCV5 | Burning effigies of corrupted politicians from other groups is a justified act |
| UCV6 | Building representative figures of corrupted politicians from other groups and destroying them in groups is a justified act |

**Table SI4**: Final Items for Diffuse Collective Violence sub-scale (Arabic - Lebanon)

| Item |  |
| --- | --- |
| DCV1 | إن ممارسة العنف الجسدي من قبل أفراد طائفتي تجاه أفراد من الطوائف الأخرى فعلٌ مُبَرّر |
| DCV2 | يمكن تبرير إستعمال الضرب من قبل أبناء طائفتي ضد أفراد من طائفة أخرى |
| DCV3 | يمكن تبرير إستعمال العنف الجسدي من قبل أبناء طائفتي ضد أفراد من طائفة أخرى |
| DCV4 | يمكن تبرير قيام أبناء طائفتي بتوجيه كلمات نابية ضد أفراد من طائفة أخرى |
| DCV5 | من المبرر أحيانًا أن يقوم أبناء طائفتي بتهديد لفظي لأفراد من الطوائف الأخرى |
| DCV6 | من المُبَرَّر أحيانًا قيام أفراد من طائفتي بإهانة لفظيّة لأفراد من طائفة أخرى على مواقع التواصل الاجتماعي |

**Table SI5**: Final Items for Upward Collective Violence sub-scale (Arabic - Lebanon)

| Item |  |
| --- | --- |
| UCV1 | يمكن تبرير الإهانة اللفظيّة الموجّهة ضد السياسيّين المنتمين إلى الطوائف الأخرى |
| UCV2 | يمكن تبرير حاجة الناس إلى توجيه العنف ضدّ زعماء بلادنا خاصة أولئك الذين ينتمون إلى الطوائف الأخرى |
| UCV3 | إن جر الدمى التي تمثل سياسيّين فاسدين من طوائف أخرى في الشوارع فعلٌ مُبرَّر |
| UCV4 | إقامة محاكمات رمزية وتعليق مشانق للدمى التي تمثّل السياسيين الفاسدين من طوائف أخرى فعلٌ مُبرَّر |
| UCV5 | حرق الدمى التي تمثّل السياسيين الفاسدين من الطوائف الأخرى فعلٌ مُبرَّر |
| UCV6 | بناء مجسمات للسياسيين الفاسدين من الطوائف الأخرى وتدميرها بشكل جماعي فعلٌ مُبرَّر |

**Table SI6**: Final Items for Diffuse Collective Violence sub-scale (Arabic - Syria)

| Item |  |
| --- | --- |
| DCV1 | إن ممارسة العنف الجسدي من قبل أفراد المعارضة السورية تجاه مؤيدي النظام فعلٌ مُبَرّر |
| DCV2 | يمكن تبرير إستعمال الضرب من قبل أفراد المعارضة السورية ضد أفراد مؤيدين للنظام |
| DCV3 | يمكن تبرير إستعمال العنف الجسدي من قبل أفراد المعارضة السورية ضد أفراد مؤيدين للنظام |
| DCV4 | يمكن تبرير قيام أفراد المعارضة السورية بتوجيه كلمات نابية ضد أفراد مؤيدين للنظام |
| DCV5 | من المبرر أحيانًا أن يقوم أفراد المعارضة السورية بتهديد لفظي لأفراد مؤيدين للنظام |
| DCV6 | من المُبَرَّر أحيانًا قيام أفراد من المعارضة السورية بإهانة لفظيّة لأفراد مؤيدين للنظام على مواقع التواصل الاجتماعي |

**Table SI7**: Final Items for Upward Collective Violence sub-scale (Arabic - Syria)

| Item |  |
| --- | --- |
| UCV1 | يمكن تبرير الإهانة اللفظيّة الموجّهة ضد السياسيّين المنتمين إلى النظام |
| UCV2 | يمكن تبرير حاجة الناس إلى توجيه العنف ضدّ زعماء بلادنا خاصة أولئك الذين ينتمون إلى النظام |
| UCV3 | إن جر الدمى التي تمثل سياسيّي النظام الفاسدين في الشوارع فعلٌ مُبرَّر |
| UCV4 | إقامة محاكمات رمزية وتعليق مشانق للدمى التي تمثّل سياسيّي النظام الفاسدين فعلٌ مُبرَّر |
| UCV5 | حرق الدمى التي تمثّل سياسيّي النظام الفاسدين فعلٌ مُبرَّر |
| UCV6 | بناء مجسمات سياسيّي النظام الفاسدين وتدميرها بشكل جماعي فعلٌ مُبرَّر |

**Table SI8**: Final Items for Diffuse Collective Violence sub-scale (Turkish)

| Item |  |
| --- | --- |
| DCV1 | Türklerin Kürtlere karşı fiziksel olarak saldırgan olması haklıdır |
| DCV2 | Türklerin Kürtlere vurması haklı olabilir |
| DCV3 | Türklerin Kürtlere fiziksel olarak zarar vermesi haklı olabilir |
| DCV4 | Türklerin Kürtlere sözel olarak hakaret etmesi haklı olabilir |
| DCV5 | Türklerin Kürtleri sözel olarak tehdit etmesi bazen haklı olabilir |
| DCV6 | Türklerin Kürtlere sosyal medyada sözel olarak hakaret etmesi bazen haklı olabilir |

**Table SI9**: Final Items for Upward Collective Violence sub-scale (Turkish)

| Item |  |
| --- | --- |
| UCV1 | Bazı Kürt siyasetçileri sözel olarak aşağılamak haklı görülebilir |
| UCV2 | İnsanların ülkemizdeki bazı Kürt siyasetçilere karşı şiddet kullanma gereksinimi haklı görülebilir |
| UCV3 | Bazı yozlaşmış Kürt siyasetçilerin temsili kuklalarını sokaklarda sürüklemek haklı bir eylemdir |
| UCV4 | Sembolik duruşmalar düzenleyip bazı  yozlaşmış Kürt siyasetçilerin temsili kuklalarını asmak haklı bir eylemdir |
| UCV5 | Bazı yozlaşmış Kürt siyasetçilerin temsili kuklalarını yakmak haklı bir eylemdir |
| UCV6 | Bazı yozlaşmış Kürt siyasetçilerin temsili figürlerini oluşturmak ve onları gruplar halinde yok etmek haklı bir eylemdir |

**Response Distributions for Collective Violence Support Items**

**Table SI10**: Distribution of chosen collective violence scale responses by item, Study 1A

| **Scale** | **Wording** | **1 (Totally**  **Disagree)** | **2** | **3** | **4** | **5**  **(Totally Agree)** | **Mean** | **SD** |
| --- | --- | --- | --- | --- | --- | --- | --- | --- |
| **Diffuse** | It is justified for members of my sect to become physically aggressive towards members of a different sect | 508  (83.83%) | 38  (6.27%0 | 37  (6.11%) | 16  (2.64%) | 7  (1.16%) | 1.31 | 0.80 |
| **Diffuse** | It can be justified for members of my sect to hit members of a different sect | 515  (84.43%) | 39  (6.39%) | 42  (6.89%) | 5  (0.82%) | 9  (1.48%) | 1.29 | 0.75 |
| **Diffuse** | It can be justified for members of my sect to physically harm members of a different sect | 515  (83.47%) | 46  (7.46%) | 33  (5.35%) | 12  (1.94%) | 11  (1.78%) | 1.31 | 0.81 |
| **Diffuse** | It is justified for members of my sect to verbally insult members of a different sect | 489  (80.03%) | 53  (8.67%) | 50  (8.18%0 | 5  (0.82%) | 14  (2.29%) | 1.37 | 0.85 |
| **Diffuse** | It is sometimes justified for members of my sect to verbally threaten members of a different sect | 418  (68.98%) | 61  (10.07%) | 73  (12.05%) | 29  (4.79%) | 25  (4.13%) | 1.65 | 1.12 |
| **Diffuse** | It can be justified for members of my sect to verbally assault members of a different sect | 474  (77.45%) | 45  (7.35%) | 62  (10.13%) | 15  (2.45%) | 16  (2.61%) | 1.45 | 0.96 |
| **Diffuse** | Destroying the property of a different sect is sometimes a justified reaction | 493  (81.35%) | 45  (7.43%) | 45  (7.43%) | 10  (1.65%) | 13  (2.15%) | 1.36 | 0.86 |
| **Diffuse** | It is justified sometimes for members of my sect to destroy property of members from a different sect | 520  (85.11%) | 36  (5.89%) | 36  (5.89%) | 6  (0.98%) | 13  (2.13%) | 1.29 | 0.80 |
| **Diffuse** | It is justified sometimes for members of my sect to damage cars of members from a different sect | 511  (84.46%) | 37  (6.12%) | 36  (5.95%) | 12  (1.98%) | 9  (1.49%) | 1.30 | 0.79 |
| **Upward** | Cursing politicians can be justified | 101  (16.5%) | 63  (10.29%) | 126  (20.59%) | 97  (15.85%) | 225  (36.76%) | 2.46 | 1.48 |
| **Upward** | Verbally insulting politicians can be justified | 118  (19.28%) | 66  (10.78%) | 113  (18.46%) | 106  (173.2%) | 209  (34.15%) | 2.36 | 1.51 |
| **Upward** | Generally, offending political leaders through drawings, falls under freedom of expression | 79  (12.80%) | 48  (7.78%) | 132  (21.39%) | 138  (22.37%) | 220  (35.66%) | 2.60 | 1.37 |
| **Upward** | Holding symbolic trials and hanging effigies representing enemy figures is a justified act | 205  (34.05%) | 76  (12.62%) | 144  (23.92%) | 72  (11.96%) | 105  (17.44%) | 1.66 | 1.48 |
| **Upward** | It is accepted that people share memes making fun of politicians they do not agree with | 102  (16.78%) | 62  (10.20%) | 137  (22.53%) | 109  (17.93%) | 198  (32.57%) | 2.39 | 1.45 |
| **Upward** | Holding symbolic trials and hanging effigies representing corrupted politicians is a justified act | 135  (22.54%) | 47  (7.85%) | 118  (19.70%) | 97  (16.19%) | 202  (33.72%) | 2.31 | 1.55 |
| **Upward** | Building representative figures of corrupted politicians and destroying them in groups is a justified act | 137  (22.50%) | 60  (9.85%) | 125  (20.53%) | 100  (16.42%) | 287  (30.71%) | 2.23 | 1.53 |
| **Upward** | Generally, people interact positively with pictures that make fun of politicians they don’t like on social media | 66  (10.84%) | 26  (4.27%) | 118  (19.38%) | 139  (22.82%) | 260  (42.69%) | 2.82 | 1.32 |

**Table SI11**: Distribution of chosen collective violence scale responses by item, Study 1B

| **Scale** | **Wording** | **1 (Totally**  **Disagree)** | **2** | **3** | **4** | **5**  **(Totally Agree)** | **Mean** | **SD** |
| --- | --- | --- | --- | --- | --- | --- | --- | --- |
| **Diffuse** | It is justified for members of my sect to become physically aggressive towards members of a different sect | 484  (83.02%) | 58  (9.95%) | 25  (4.29%) | 9  (1.54%) | 7  (1.20%) | 1.28 | 0.72 |
| **Diffuse** | It can be justified for members of my sect to hit members of a different sect | 489  (83.45%) | 66  (11.26%) | 23  (3.92%) | 2  (0.34%) | 6  (1.03%) | 1.24 | 0.64 |
| **Diffuse** | It can be justified for members of my sect to physically harm members of a different sect | 492  (83.82%) | 61  (10.39%) | 23  (3.92%) | 5  (0.85%) | 6  (1.02%) | 1.25 | 0.66 |
| **Diffuse** | It is justified for members of my sect to verbally insult members of a different sect | 434  (73.56%) | 89  (15.08%) | 43  (7.29%) | 10  (1.69%) | 14  (2.38%) | 1.44 | 0.88 |
| **Diffuse** | It is sometimes justified for members of my sect to verbally threaten members of a different sect | 418  (71.09%) | 89  (15.14%) | 52  (8.84%) | 16  (2.72%) | 13  (2.21%) | 1.50 | 0.93 |
| **Diffuse** | It is sometimes justified for members of my sect to verbally insult members of a different sect on social media | 432  (73.22%) | 90  (15.25%) | 51  (8.64%) | 10  (1.69%) | 7  (1.20%) | 1.42 | 0.81 |
| **Diffuse** | Destroying the property of a different sect is sometimes a justified reaction | 463  (78.74%) | 63  (10.71%) | 40  (6.80%) | 8  (1.36%) | 14  (2.39%) | 1.38 | 0.86 |
| **Diffuse** | Burning cars of members from a different sect can be a justified act | 509  (85.25%) | 50 (8.38%) | 30  (5.03%) | 4  (0.67%) | 4  (0.67%) | 1.23 | 0.63 |
| **Diffuse** | It is justified sometimes for members of my sect to damage cars of members from a different sect | 487  (82.26%) | 59 (9.97%) | 31  (5.24%) | 5  (0.84%) | 10  (1.69%) | 1.30 | 0.75 |
| **Upward** | Verbally insulting politicians can be justified | 56  (9.51%) | 51  (8.66%) | 78  (13.24%) | 106  (18.00%) | 298  (50.59%) | 2.92 | 1.36 |
| **Upward** | One can justify people’s need to be violent towards our country’s leaders | 51  (8.49%) | 55  (9.15%) | 69  (11.48%) | 142  (23.63%) | 284  (47.25%) | 2.92 | 1.31 |
| **Upward** | Dragging effigies representing corrupted politicians in the streets is a justified act | 67  (11.4%) | 55  (9.35%) | 87  (14.80%) | 96  (16.32%) | 283  (48.13%) | 2.80 | 1.41 |
| **Upward** | Insulting those responsible for our situation is a justified act | 58  (9.76%) | 36  (6.06%) | 68  (11.45%) | 112  (18.86%) | 320  (53.87%) | 3.01 | 1.33 |
| **Upward** | Destroying property of corrupted politicians can be justified | 76  (12.92%) | 45  (7.65%) | 78  (13.27%) | 96  (16.33%) | 293  (49.83%) | 2.82 | 1.44 |
| **Upward** | Holding symbolic trials and hanging effigies representing corrupted politicians is a justified act | 71  (11.90%) | 48  (8.04%) | 95  (15.91%) | 92  (15.41%) | 291  (48.74%) | 2.81 | 1.42 |
| **Upward** | Being verbally violent against those who got us here is a justified act | 55  (9.19%) | 40  (6.69%) | 73  (12.21%) | 109  (18.23%) | 321  (53.68%) | 3.01 | 1.33 |
| **Upward** | Burning effigies of corrupted politicians is a justified act | 51  (8.59%) | 48  (8.12%) | 89  (15.06%) | 99  (16.75%) | 304  (51.48%) | 2.94 | 1.33 |
| **Upward** | Building representative figures of corrupted politicians and destroying them in groups is a justified act | 88  (14.83%) | 69  (11.64%) | 113  (19.06%) | 89  (15.01%) | 234  (39.46%) | 2.53 | 1.47 |

**Table SI12**: Distribution of collective violence scale responses by item and experimental condition, Study 2. Cn=Condition (C=Control, Str=Strong Threat Prime, Sub=Subtle Threat Prime).

| **Scale** | **Wording** | **Cn** | **1 (Totally**  **Disagree)** | **2** | **3** | **4** | **5**  **(Totally Agree)** | **Mean** | **SD** |
| --- | --- | --- | --- | --- | --- | --- | --- | --- | --- |
| **Diffuse** | It is justified for members of my ingroup to become physically aggressive towards members of an outgroup | C | 237  (70.96) | 40  (11.98%) | 28  (8.38%) | 13  (3.89%) | 16  (4.79%) | 1.60 | 1.10 |
|  |  | Str | 256  (79.75%) | 29  (9.03%) | 16  (4.98%) | 9 (2.8%) | 11 (3.43%) | 1.41 | 0.96 |
|  |  | Sub | 274  (77.18%) | 30  (8.45%) | 24 (6.76%) | 7  (1.97%) | 20  (5.63%) | 1.50 | 1.08 |
| **Diffuse** | It can be justified for members of my ingroup to hit members of an outgroup | C | 232  (68.44%) | 36  (10.62%) | 37  (10.91%) | 12  (3.54%) | 22  (6.49%) | 1.69 | 1.19 |
|  |  | Str | 257  (80.06%) | 27  (8.41%) | 13  (4.05%) | 8  (2.49%) | 16  (4.98%) | 1.44 | 1.04 |
|  |  | Sub | 286  (79.89%) | 29  (8.1%) | 23  (6.42%) | 9  (2.51%) | 11 (3.07%) | 1.41 | 0.94 |
| **Diffuse** | It can be justified for members of my ingroup to physically harm members of an outgroup | C | 225  (67.16%) | 31  (9.25%) | 45  (13.43%) | 16  (4.78%) | 18  (5.37%) | 1.72 | 1.26 |
|  |  | Str | 254  (79.13%) | 26  (8.10%) | 18  (5.61%) | 6  (1.87%0 | 17  (5.30%) | 1.46 | 1.10 |
|  |  | Sub | 281  (79.60%) | 28  (7.93%) | 27  (7.65%) | 5  (1.42%0 | 12  (3.40%) | 1.41 | 1.08 |
| **Diffuse** | It is justified for members of my ingroup to verbally insult members of an outgroup | C | 212  (63.66%) | 41  (12.31%) | 38  (11.41%) | 17  (5.11%) | 25  (7.51%) | 1.80 | 1.33 |
|  |  | Str | 250  (76.92%) | 30  (9.23%) | 18  (5.54%) | 8  (2.46%) | 19  (5.85%) | 1.51 | 1.15 |
|  |  | Sub | 259  (70.96%) | 40  (10.96%) | 40  (10.96%0 | 9  (2.47%) | 17  (4.66%) | 1.59 | 1.10 |
| **Diffuse** | It is sometimes justified for members of my ingroup to verbally threaten members of an outgroup | C | 198  (60.55%) | 36  (11.01%) | 45  (13.76%) | 20  (6.12%) | 28  (8.56%) | 1.91 | 1.17 |
|  |  | Str | 236  (72.84%) | 35  (10.8%) | 20  (6.17%) | 14  (4.32%) | 19  (5.86%) | 1.60 | 0.98 |
|  |  | Sub | 245  (69.41%) | 42  (11.90%) | 38 (10.76%) | 12  (3.40%) | 16  (4.53%) | 1.62 | 1.15 |
| **Diffuse** | It is sometimes justified for members of my ingroup to verbally insult members of an outgroup on social media | C | 219  (6.96%) | 46  (13.86%) | 32  (9.64%) | 17  (5.12%) | 18  (5.42%) | 1.70 | 1.42 |
|  |  | Str | 250  (76.22%) | 39  (11.89%) | 19  (5.79%) | 7  (2.13%) | 13  (3.96%) | 1.46 | 1.45 |
|  |  | Sub | 255  (71.23%) | 33  (9.22%) | 38  (10.61%) | 12  (3.35) | 20  (5.59%) | 1.63 | 1.49 |
| **Upward** | Verbally insulting politicians from other groups can be justified | C | 171  (51.51%) | 49  (14.76%) | 50  (15.06%) | 21  (6.33%) | 41  (12.35%) | 2.13 | 1.45 |
|  |  | Str | 149  (46.13%) | 41  (12.69%) | 65  (20.12%) | 25  (7.74%) | 43  (13.31%) | 2.29 | 1.43 |
|  |  | Sub | 140  (38.89%) | 50  (13.89%) | 75  (20.83%) | 35  (9.72%) | 60  (16.67%) | 2.51 | 1.46 |
| **Upward** | One can justify people’s need to be violent towards our country’s leaders especially those from other groups | C | 163  (49.1%) | 39  (11.75%) | 61  (18.37%) | 27  (8.13%) | 42  (12.65%) | 2.23 | 1.43 |
|  |  | Str | 117  (36.45%) | 48  (14.95%) | 80  (24.92%) | 29  (9.03%) | 47  (14.64%) | 2.50 | 1.57 |
|  |  | Sub | 126  (35.00%) | 48  (13.33%) | 95  (26.39%) | 30  (8.33%) | 61  (16.94%) | 2.59 | 1.62 |
| **Upward** | Dragging effigies representing corrupted politicians from other sects in the streets is a justified act | C | 159  (48.04%) | 42  (12.69%) | 62  (18.73%) | 28  (8.46%) | 40  (12.08%) | 2.24 | 1.49 |
|  |  | Str | 121  (37.46%) | 36  (11.15) | 66  (20.43%) | 29  (8.98%) | 71  (21.98%) | 2.67 | 1.57 |
|  |  | Sub | 130  (36.31%) | 41  (11.45%) | 61  (17.04%) | 36  (10.06%) | 90  (25.14%) | 2.76 | 1.60 |
| **Upward** | Holding symbolic trials and hanging effigies representing corrupted politicians from other groups is a justified act | C | 154  (45.83%) | 51  (15.18%) | 53  (15.77%) | 26  (7.74%) | 52  (15.48%) | 2.31 | 1.49 |
|  |  | Str | 85  (26.48%) | 37  (11.53%) | 70  (21.81%) | 35  (10.90%) | 94  (29.28%) | 3.05 | 1.57 |
|  |  | Sub | 94  (26.26%) | 42  (11.73%) | 65  (18.16%) | 41  (11.45%) | 116  (32.4%) | 3.12 | 1.60 |
| **Upward** | Burning effigies of corrupted politicians from other groups is a justified act | C | 142  (43.03%) | 42  (12.73%) | 67  (20.3%) | 27  (8.18%) | 52  (15.76%) | 2.41 | 1.49 |
|  |  | Str | 100  (31.15%) | 33  (10.28%) | 76  (23.68%) | 25  (7.79) | 87  (27.10%) | 2.89 | 1.58 |
|  |  | Sub | 120  (33.71%) | 38  (10.67) | 62  (17.42%) | 45  (12.64%) | 91  (25.56%) | 2.86 | 1.61 |
| **Upward** | Building representative figures of corrupted politicians from other groups and destroying them in groups is a justified act | C | 158  (46.75%) | 58  (17.16%) | 55  (16.27%) | 18  (5.33%) | 49  (14.5%) | 2.24 | 1.45 |
|  |  | Str | 107  (33.23%) | 36  (11.18%) | 64  (19.88%) | 40  (12.42%) | 75  (23.29%) | 2.81 | 1.57 |
|  |  | Sub | 108  (30.08%) | 44  (12.26%) | 74  (20.61%) | 36  (10.03%) | 97  (27.02%) | 2.92 | 1.58 |

**Table SI13**: Distribution of collective violence scale responses by item and experimental condition, Study 3 Cn=Condition (C=Control, Str=Strong Threat Prime, Sub=Subtle Threat Prime).

| **Scale** | **Wording** | **Cn** | **1 (Totally**  **Disagree)** | **2** | **3** | **4** | **5**  **(Totally Agree)** | **Mean** | **SD** |
| --- | --- | --- | --- | --- | --- | --- | --- | --- | --- |
| **Diffuse** | It is justified for the opposition to become physically aggressive towards regime supporters | C | 108  (60.67%) | 23  (12.92%) | 17  (9.55%) | 11  (6.18%) | 19  (10.67%) | 1.93 | 1.38 |
|  |  | Str | 109  (54.5%) | 33  (16.50%) | 24  (12.00%) | 12  (6.00%) | 22  (11.00%) | 2.03 | 1.38 |
|  |  | Sub | 87  (53.37%) | 28  (17.18%) | 20  (12.27%) | 7  (4.29%) | 21  (12.88%) | 2.06 | 1.41 |
| **Diffuse** | It can be justified for the opposition to hit regime supporters | C | 105  (58.66%) | 30  (16.676%) | 16  (8.94%) | 10  (5.59%) | 18  (10.06%) | 1.92 | 1.34 |
|  |  | Str | 115  (56.37%) | 35  (17.16%) | 28  (13.73%) | 6  (2.94%) | 20  (9.8%) | 1.93 | 1.31 |
|  |  | Sub | 81  (50.00%) | 40  (24.69%) | 11  (6.79%) | 6  (3.70%) | 24  (14.81%) | 2.09 | 1.43 |
| **Diffuse** | It can be justified for the opposition to physically harm regime supporters | C | 98  (55.37%) | 30  (16.95%) | 22  (12.43%) | 4  (2.26%) | 23  (12.99%) | 2.01 | 1.40 |
|  |  | Str | 105  (52.24%) | 41  (20.4%) | 22  (10.95%) | 12  (5.97%) | 21  (10.45%) | 2.02 | 1.35 |
|  |  | Sub | 92  (57.50%) | 25  (15.62%) | 15  (9.38%) | 5  (3.12%) | 23  (14.37%) | 2.01 | 1.45 |
| **Diffuse** | It is justified for the opposition to verbally insult regime supporters | C | 78  (43.09%) | 40  (22.1%) | 29  (16.02%) | 13  (7.18%) | 21  (11.60%) | 2.22 | 1.37 |
|  |  | Str | 76  (37.81%) | 47  (23.38%) | 25  (12.44%) | 21  (10.45%) | 32  (15.92%) | 2.43 | 1.48 |
|  |  | Sub | 75  (46.58%) | 28  (17.39%) | 22  (13.66%0 | 11  (6.83%) | 25  (15.53%) | 2.27 | 1.49 |
| **Diffuse** | It is sometimes justified for the opposition to verbally threaten regime supporters | C | 106  (58.89%) | 18  (10.00%) | 18  (10.00%) | 18  (10.00%) | 20  (11.11%) | 2.04 | 1.45 |
|  |  | Str | 111  (54.68%) | 31  (15.27%) | 33  (16.26%) | 5  (2.46%) | 23  (11.33%) | 2.00 | 1.35 |
|  |  | Sub | 69  (42.86%) | 37  (22.98%) | 18  (11.18%) | 10  (6.21%) | 27 (16.77%) | 2.31 | 1.49 |
| **Diffuse** | It is sometimes justified for the opposition to verbally insult regime supporters on social media | C | 76  (42.70%) | 38  (21.35%) | 25  (14.04%) | 17  (9.55%) | 22  (123.6%) | 2.27 | 1.41 |
|  |  | Str | 73  (36.14%) | 43  (21.29%) | 31  (15.35%) | 21  (10.40%) | 34  (16.83%) | 2.50 | 1.48 |
|  |  | Sub | 77  (47.53%) | 30  (18.52%) | 16  (9.88%) | 16  (9.88%) | 23  (14.20%) | 2.25 | 1.48 |
| **Upward** | Verbally insulting regime politicians can be justified | C | 41  (22.91%) | 20  (11.17%) | 42  (23.46%) | 34  (18.99%) | 42  (23.46%) | 3.09 | 1.47 |
|  |  | Str | 42  (20.79%) | 18  (8.91%) | 56  (27.72%) | 32  (15.84%) | 54  (26.73%) | 3.19 | 1.46 |
|  |  | Sub | 37  (22.84%) | 26  (16.05%) | 33  (20.37%) | 30  (18.52%) | 36  (22.22%) | 3.01 | 1.41 |
| **Upward** | One can justify people’s need to be violent towards our country’s leaders especially those from the regime | C | 40  (22.35%) | 25  (13.97%) | 44  (24.58%) | 29  (16.20%) | 41  (22.91%) | 3.03 | 1.46 |
|  |  | Str | 39  (19.5%) | 19  (9.50%) | 62  (31.0%) | 37  (18.5%) | 43  (21.50%) | 3.13 | 1.38 |
|  |  | Sub | 35  (21.21%) | 16  (9.70%) | 50  (30.30%) | 28  (16.97%) | 36  (21.82%) | 3.08 | 1.41 |
| **Upward** | Dragging effigies representing corrupted regime politicians in the streets is a justified act | C | 34  (18.78%) | 19  (10.5%) | 49  (27.07%) | 37  (20.44%) | 42  (23.2%) | 3.19 | 1.40 |
|  |  | Str | 39  (19.90%) | 16  (8.16%) | 56  (28.57%) | 39  (19.90%) | 46  (23.47%) | 3.19 | 1.41 |
|  |  | Sub | 33  (20.37%) | 16  (9.88%) | 35  (21.60%) | 34  (20.99%) | 44  (27.16%) | 3.25 | 1.47 |
| **Upward** | Holding symbolic trials and hanging effigies representing corrupted regime politicians is a justified act | C | 32  (18.29%) | 22  (12.57%) | 40  (22.86%) | 37  (21.14%) | 44  (25.14%) | 3.22 | 1.43 |
|  |  | Str | 39  (19.50%) | 14  (7.00%) | 49  (24.50%) | 44  (22.00%) | 54  (27.00%) | 3.30 | 1.44 |
|  |  | Sub | 33  (20.25%) | 15  (9.20%) | 31  (19.02%) | 37  (22.70%) | 47  (28.83%) | 3.31 | 1.48 |
| **Upward** | Burning effigies of corrupted regime politicians is a justified act | C | 31  (17.22%) | 21  (11.67%) | 50  (27.78%) | 34  (18.89%) | 44  (24.44%) | 3.21 | 1.39 |
|  |  | Str | 39  (19.31%) | 16  (7.92%) | 59  (29.21%) | 36  (17.82%) | 52  (25.74%) | 3.23 | 1.42 |
|  |  | Sub | 32  (19.51%) | 16  (9.76%) | 35  (21.34%) | 34  (20.73%) | 47  (28.66%) | 3.29 | 1.47 |
| **Upward** | Building representative figures of corrupted regime politicians and destroying them in groups is a justified act | C | 27  (15.43%) | 25  (14.29%) | 50  (28.57%) | 27  (15.43%) | 46  (26.29%) | 3.23 | 1.39 |
|  |  | Str | 40  (19.7%) | 16  (7.88%) | 67  (33.00%) | 36  (17.73%) | 44  (21.67%) | 3.14 | 1.38 |
|  |  | Sub | 29  (17.58%) | 16  (9.70%) | 45  (27.27%) | 27  (16.36%) | 48  (29.09%) | 3.30 | 1.43 |

**Table SI14**: Distribution of collective violence scale responses by item, Study 4

| **Scale** | **Wording** | **1 (Totally**  **Disagree)** | **2** | **3** | **4** | **5**  **(Totally Agree)** | **Mean** | **SD** |
| --- | --- | --- | --- | --- | --- | --- | --- | --- |
| **Diffuse** | It is justified for Turks to be physically aggressive towards Kurds. | 147  (75.77%) | 17  (8.76%) | 20  (10.31%) | 8  (4.12%) | 2  (1.03%) | 1.46 | 0.91 |
| **Diffuse** | It may be justified for Turks to hit Kurds. | 150  (77.32%) | 19  (9.79%) | 17  (8.76%) | 7  (3.61%) | 1  (0.52%) | 1.40 | 0.84 |
| **Diffuse** | It may be justified for Turks to physically harm Kurds. | 150  (77.32%) | 17  (8.76%) | 19  (9.79%) | 7  (3.61%) | 1  (0.52%) | 1.41 | 0.85 |
| **Diffuse** | It may be justified for Turks to verbally insult Kurds. | 134  (69.07%) | 26  (13.4%) | 22  (11.34%) | 12  (6.19%) | 0  (0.00%) | 1.55 | 0.92 |
| **Diffuse** | It may sometimes be justified for Turks to verbally threaten Kurds. | 123  (63.4%) | 32  (16.49%) | 22  (11.34%) | 16  (8.25%) | 1  (0.52%) | 1.66 | 1.01 |
| **Diffuse** | It may sometimes be justified for Turks to verbally insult Kurds on social media. | 122  (62.89%) | 32  (16.49%) | 24  (12.37%) | 16  (8.25%) | 0  (0.00%) | 1.66 | 0.99 |
| **Diffuse** | Destroying the properties of Kurds is sometimes a justified reaction. | 154  (79.38%) | 20  (10.31%) | 12  (6.19%) | 7  (3.61%) | 1  (0.52%) | 1.36 | 0.80 |
| **Diffuse** | Burning the cars of Kurds may be a justifiable action. | 167  (86.08%) | 9  (4.64%) | 13  (6.7%) | 4  (2.06%) | 1  (0.52%) | 1.26 | 0.72 |
| **Diffuse** | It is sometimes justified for Turks to damage the cars of Kurds. | 164  (84.54%) | 9  (4.64%) | 14  (7.22%) | 6  (3.09%) | 1  (0.52%) | 1.30 | 0.78 |
| **Upward** | Verbally insulting some Kurdish politicians can be justified. | 91  (46.91%) | 37  (19.07%) | 29  (14.95%) | 31  (15.98%) | 6  (3.09%) | 2.09 | 1.23 |
| **Upward** | One can justify people’s need to be violent towards some Kurdish politicians | 121  (62.37%) | 29  (14.95%) | 21  (10.82%) | 17  (8.76%0 | 6  (3.09%) | 1.75 | 1.14 |
| **Upward** | Insulting some Kurdish politicians who are responsible for our situation is a justified act | 79  (40.72%) | 24  (12.37%) | 34  (17.53%) | 51  (26.29%) | 6  (3.09%) | 2.39 | 1.33 |
| **Upward** | Dragging effigies representing some corrupted Kurdish politicians in the streets is a justified act | 120  (61.86%) | 21  (10.82%) | 18  (9.28%) | 29  (14.95%) | 6  (3.09%) | 1.87 | 1.26 |
| **Upward** | Destroying properties of some corrupted Kurdish politicians can be justified | 124  (63.92%) | 21  (10.82%) | 23  (11.86%) | 22  (11.34%) | 4  (2.06%) | 1.77 | 1.16 |
| **Upward** | Holding symbolic trials and hanging effigies representing some corrupted Kurdish politicians is a justified act | 127  (65.46%) | 22  (11.34%) | 20 190.31%) | 18  (9.28%) | 7  (3.61%) | 1.74 | 1.18 |
| **Upward** | Being verbally violent against some Kurdish politicians who got us here is a justified act | 78  (40.21%) | 29  (14.95%) | 30  (15.46%) | 46  (23.71%) | 11  (5.67%) | 2.40 | 1.37 |
| **Upward** | Burning effigies of some corrupted Kurdish politicians is a justified act | 126  (64.95%) | 24  (12.37%) | 23  (11.86%) | 13  (6.70%) | 8  (4.12%) | 1.73 | 1.16 |
| **Upward** | Building representative figures of some corrupted Kurdish politicians and destroying them in groups is a justified act | 124  (63.92%) | 24  (12.37%) | 25  (12.89%) | 14  (7.22%) | 7  (3.61%) | 1.74 | 1.15 |

**Factor Loadings and Model Fit Using Oblimin Rotation**

| **Table SI15** |  |  |  |
| --- | --- | --- | --- |
|  |  |  |  |
| *Factor loadings of the final items used in measuring collective violence, Study 1A (Ordered, Oblimin Rotation)* | |  |  |
|  |  | 1 | 2 |
| 7. | It is justified for members of my sect to become physically aggressive towards members of a different sect | **0.910** | -0.058 |
| 1. | It can be justified for members of my sect to hit members of a different sect | **0.949** | -0.062 |
| 3. | It can be justified for members of my sect to physically harm members of a different sect | **0.907** | -0.124 |
| 8. | It is justified for members of my sect to verbally insult members of a different sect | **0.914** | 0.102 |
| 12. | It is sometimes justified for members of my sect to verbally threaten members of a different sect | **0.838** | 0.121 |
| 13. | It can be justified for members of my sect to verbally assault members of a different sect | **0.884** | 0.150 |
| 36. | Destroying the property of a different sect is sometimes a justified reaction | **0.861** | 0.006 |
| 37. | It is justified sometimes for members of my sect to destroy property of members from a different sect | **0.878** | -0.067 |
| 40. | It is justified sometimes for members of my sect to damage cars of members from a different sect | **0.876** | -0.041 |
| 10. | Cursing politicians can be justified | -0.092 | **0.785** |
| 14. | Verbally insulting politicians can be justified | -0.058 | **0.771** |
| 20. | Generally, offending political leaders through drawings, falls under freedom of expression | -0.157 | **0.710** |
| 28. | Holding symbolic trials and hanging effigies representing enemy figures is a justified act | 0.205 | **0.708** |
| 29. | It is accepted that people share memes making fun of politicians they do not agree with | -0.024 | **0.779** |
| 32. | Holding symbolic trials and hanging effigies representing corrupted politicians is a justified act | 0.037 | **0.820** |
| 33. | Building representative figures of corrupted politicians and destroying them in groups is a justified act | 0.136 | **0.804** |
| 35. | Generally, people interact positively with pictures that make fun of politicians they don’t like on social media | -0.124 | **0.626** |
|  |  |  |  |
| Note: Pattern factor matrix, Rotation Method: Oblimin. | |  |  |
| Factor loading characteristic of specific factors are indicated in bold | |  |  |

| **Table SI16** |  |  |  |
| --- | --- | --- | --- |
|  |  |  |  |
| *Factor loadings of the final items included in the Two-Dimension Collective Violence Beliefs Scale (CVBS: 2D), Study 1B (Ordinal Measures, Oblimin Rotation)* | |  |  |
|  |  | 1 | 2 |
| 1. | It is justified for members of my sect to become physically aggressive towards members of a different sect | **0.895** | -0.041 |
| 2. | It can be justified for members of my sect to hit members of a different sect | **0.893** | 0.081 |
| 3. | It can be justified for members of my sect to physically harm members of a different sect | **0.868** | 0.046 |
| 4. | It is justified for members of my sect to verbally insult members of a different sect | **0.841** | 0.047 |
| 5. | It is sometimes justified for members of my sect to verbally threaten members of a different sect | **0.836** | 0.033 |
| 6. | It is sometimes justified for members of my sect to verbally insult members of a different sect on social media | **0.849** | -0.015 |
| 7. | Destroying the property of a different sect is sometimes a justified reaction | **0.732** | 0.087 |
| 8. | Burning cars of members from a different sect can be a justified act | **0.829** | -0.027 |
| 9. | It is justified sometimes for members of my sect to damage cars of members from a different sect | **0.836** | 0.002 |
| 10. | Verbally insulting politicians can be justified | -0.024 | **0.880** |
| 11. | One can justify people’s need to be violent towards our country’s leaders | 0.055 | **0.839** |
| 12. | Dragging effigies representing corrupted politicians in the streets is a justified act | -0.023 | **0.878** |
| 20. | Insulting those responsible for our situation is a justified act | 0.012 | **0.824** |
| 24. | Destroying property of corrupted politicians can be justified | 0.056 | **0.811** |
| 27. | Holding symbolic trials and hanging effigies representing corrupted politicians is a justified act | -0.013 | **0.896** |
| 29. | Being verbally violent against those who got us here is a justified act | -0.056 | **0.857** |
| 30. | Burning effigies of corrupted politicians is a justified act | -0.037 | **0.938** |
| 31. | Building representative figures of corrupted politicians and destroying them in groups is a justified act | 0.066 | **0.837** |
|  |  |  |  |
| Note: Pattern factor matrix, Rotation Method: Varimax with Kaiser Normalisation. | |  |  |
| Factor loading characteristic of specific factors are indicated in bold | |  |  |
